# Macrophage-mediated antibiotic evasion and competitive dominance of *mcr-3*-carrying *Escherichia coli*

**DOI:** 10.1371/journal.ppat.1014427

**Published:** 2026-07-14

**Authors:** Shuyu Tan, Wenjuan Yin, Jian Sun, Wenguang Xiong, Yina An, Lu Qiao, Pu Zhang, Siqi Sun, Qianru Li, Leiming Fu, Yifan Liu, Yaqian Xue, Shuyi Huang, Yixi Liu, Yang Wang, Yanjun Dong

**Affiliations:** 1 State Key Laboratory of Veterinary Public Health and Safety, College of Veterinary Medicine, China Agricultural University, Beijing, China; 2 College of Basic Medical Science, Key Laboratory of Pathogenesis Mechanism and Control of Inflammatory-Autoimmune Diseases of Hebei Province, Hebei University, Baoding, China; 3 State Key Laboratory of Animal Disease Control and Prevention, College of Veterinary Medicine, South China Agricultural University, Guangzhou, China; 4 Guangdong Provincial Key Laboratory of Veterinary Pharmaceutics Development and Safety Evaluation, College of Veterinary Medicine, South China Agricultural University, Guangzhou, China; 5 The Key Laboratory of Medical Molecular Cell Biology of Shanxi Province, Institutes of Biomedical Sciences, Shanxi University, Taiyuan, China; University of Virginia Health System, UNITED STATES OF AMERICA

## Abstract

Mobile colistin resistance (*mcr*) genes compromise the efficacy of last-resort polymyxin antibiotics. Notably, the global prevalence of *mcr-3* has continued to increase despite reductions in colistin use, suggesting that selective forces beyond direct antibiotic pressure contribute to its persistence. Here, *mcr-3*-positive *Escherichia coli* is shown to confer a survival advantage by reprogramming macrophage immunity. MCR-3-mediated lipid A modification blunted TLR4-NF-κB signaling, suppressed macrophage reactive oxygen species generation, and delayed phagosome-lysosome fusion, allowing *mcr-3*-positive strains to evade intracellular killing. Integrated transcriptomic and metabolomic analyses revealed extensive immunometabolic rewiring in infected macrophages, including altered glycerophospholipid and energy metabolism. Consistently, *mcr-3* enhanced bacterial tolerance to ferrous iron stress, likely mitigating host-induced ferroptotic damage. In a mouse co-infection model, *mcr-3*-positive strains outcompeted isogenic negative strains under antibiotic treatment without differing in *in vitro* susceptibility, indicating that immune evasion rather than intrinsic drug resistance alone drives their competitive advantage. These findings reveal a dual mechanism where *mcr-3* confers both antimicrobial resistance and immune suppression, enabling persistence under antibiotic pressure and highlighting the threat of *mcr-3* dissemination independent of polymyxin use.

## Author summary

Mobile colistin resistance genes reduce the effectiveness of polymyxins, which are often used as last-line antibiotics. Among them, *mcr-3* has continued to increase globally even after colistin use declined. This trend suggests that *mcr-3* may provide advantages beyond antibiotic resistance. In this study, we investigated whether *mcr-3* helps bacteria survive interactions with the host immune system. We found that bacteria carrying *mcr-3* are better able to persist inside macrophages, which are immune cells responsible for engulfing and killing invading microbes. In animal models treated with antibiotics, these bacteria outcompeted closely related strains lacking the gene, despite having similar drug susceptibility under laboratory conditions. These results indicate that *mcr-3* rewires macrophage immunity to couple antibiotic survival with intracellular persistence. This dual function may help explain why *mcr-3* continues to disseminate even in environments where polymyxin use has declined, emphasizing the need to consider host-pathogen interactions when evaluating the spread of antibiotic resistance genes.

## Introduction

The global spread of mobile colistin resistance genes (*mcr*) poses a serious threat to public health, undermining the efficacy of last-line polymyxin therapy [1,2]. Colistin exerts its bactericidal activity by electrostatically binding to the negatively charged phosphate groups at the 1 and 4’ positions of lipid A in lipopolysaccharide, thereby destabilizing the outer membrane and leading to membrane disruption. MCR enzymes, including MCR-3, reduce colistin susceptibility by transferring phosphoethanolamine (PEtN) to lipid A. This PEtN modification partially neutralizes the negative charge of the bacterial outer membrane, thereby decreasing colistin binding and attenuating colistin-mediated membrane disruption [3]. Since the first report of *mcr-1* in 2015, ten *mcr* variants (*mcr-1* to *mcr-10*) have been identified across human, animal, food, and environmental sources, with *mcr-1*, *mcr-3*, and *mcr-9* being the most prevalent worldwide [3–6]. Following the banning of colistin as a livestock growth promoter, a notable decline in *mcr-1* detection has been documented in many regions [7,8]. However, in contrast, the global prevalence of *mcr-3* has paradoxically continued to rise across environmental, livestock, and clinical sectors [9]. In China, *mcr-3* detection even increased between 2016 and 2019 despite the colistin ban [9,10], suggesting that forces beyond direct colistin selection are driving the success of *mcr-3*-harboring strains. These trends indicate that colistin pressure alone cannot fully explain the epidemiological dominance of *mcr-3* strains.

Mechanistic studies have provided a partial explanation for this discrepancy. Previous work demonstrated that unlike MCR-1, whose expression incurs a fitness cost by compromising membrane integrity, MCR-3 imposes minimal burden on bacterial growth, likely due to differences in mRNA stability and protein expression that prevent excessive membrane stress [9]. Nevertheless, the persistence of resistant bacteria in real-world ecosystems cannot be attributed solely to fitness costs. In clinical and agricultural settings, antibiotic treatment creates a dynamic interface between pathogens, antimicrobial agents, and host immune defenses. Within this complex environment, bacterial survival and expansion are driven not only by antibiotic selection but also by host immunity and microbial competition [11,12]. Phagocyte-mediated clearance is central to this process: strains that evade phagocytic killing are more likely to survive, colonize, and disseminate, particularly when antibiotic exposure is suboptimal [13].

Our previous findings revealed that *mcr-3*-positive *Escherichia coli* (*E. coli*) were less susceptible to macrophage phagocytosis than *mcr-3*-negative strains, suggesting an early advantage in immune evasion [14]. However, it remains unclear whether these strains can persist intracellularly and withstand macrophage killing under antibiotic pressure. Given the continued global rise of *mcr-3* concurrent with the decline of *mcr-1* [9] and evidence that typically extracellular bacteria can survive within host cells under specific conditions [15], we hypothesize that *mcr-3* may provide benefits beyond colistin resistance by promoting intracellular persistence and immune evasion, even during antibiotic challenge.

Macrophages eliminate bacterial pathogens through multiple mechanisms, including the generation of reactive oxygen species (ROS), lysosomal degradation within phagolysosomes [16], and iron-dependent oxidative bursts, a process linked to ferroptosis [17]. However, many pathogens have evolved strategies to subvert these defenses. For instance, *Yersinia* can modulate host cell death pathways; *Mycobacterium tuberculosis* interferes with phagosome maturation; and *Staphylococcus aureus* suppresses macrophage ROS generation [16,18]. We recently revealed that *mcr-3*-positive *E. coli* are less susceptible to macrophage phagocytosis than *mcr-3*-negative bacteria [14], suggesting an early immune evasion advantage. It remained unknown, however, whether *mcr-3* also enables *E. coli* to survive and replicate within macrophages—especially under antibiotic pressure.

Given the continued spread of *mcr-3* despite reduced colistin use, we assumed that *mcr-3* provides advantages beyond colistin resistance by promoting intracellular persistence and immune evasion. To test this, we examined the interaction of *mcr-3*-positive *E. coli* with macrophages under antibiotic pressure using both *in vitro* infection assays and *in vivo* co-infection models. We found that *mcr-3*-positive strains consistently outcompeted isogenic *mcr-3*-negative counterparts, suggesting enhanced intracellular survival advantage. Integrated transcriptomic and metabolomic analyses further revealed that *mcr-3*-positive *E. coli* infection drives a pronounced reprogramming of macrophage metabolism and immune signaling compared with *mcr-3*-negative strains. Key endogenous antimicrobial defenses were attenuated: ROS production was suppressed, phagolysosomal maturation was impaired, and the bacteria exhibited increased tolerance to ferrous iron stress. Collectively, our findings demonstrate that *mcr-3* allows *E. coli* to evade host killing and persist during antibiotic treatment, which likely contributes to its sustained dissemination across diverse environments.

## Results

### *mcr-3* confers a competitive advantage to *E. coli* under antibiotic pressure

**Fig 1:**
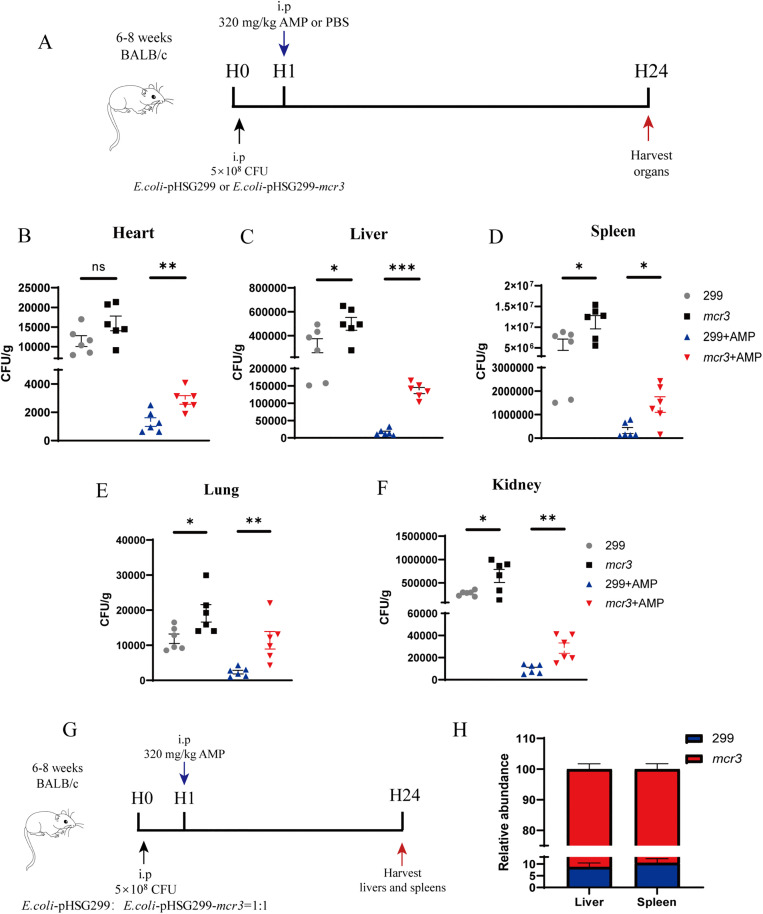
The *mcr-3* gene confers a competitive advantage to *E. coli* in macrophage-associated infections. **(A)** Schematic of mouse infection models. BALB/c mice were intraperitoneally inoculated with either *E. coli*-pHSG299 or *E. coli*-pHSG299-*mcr-3*, followed 1 h later by ampicillin (AMP) or PBS treatment. At 24 h, bacterial burdens in heart, liver, spleen, lung, and kidney were determined by plating tissue homogenates. (n = 6 mice per group). **(B-F)** Bacterial loads (CFU/g) in indicated organs from single-strain infections. Data points represent six mice. **(G)** Schematic of mouse competitive co-infection model. BALB/c mice were intraperitoneally co-inoculated with a 1:1 mixture of *E. coli*-pHSG299 and *E. coli*-pHSG299-*mcr-3*, followed 1 h later by AMP treatment. At 24 h, strain composition in the liver and spleen was determined by PCR screening of colonies. For each organ of each mouse, 100 colonies were randomly selected (n = 6 mice per group). **(H)** Relative abundance of *mcr-3*-positive and -negative strains recovered from the liver and spleen of co-infected mice at 24 h post-infection. Data are shown as the mean ± SEM. Multiple t tests was conducted for pairwise comparisons. *P < 0.05, **P < 0.01, ***P ≤ 0.001; ns, not significant.

To investigate whether *mcr-3* provides benefits beyond colistin resistance, we examined its impact under treatment with antibiotics commonly used for extracellular bacterial clearance in animal models—ampicillin (AMP) and gentamicin. Despite similar MICs between *mcr-3*-positive and *mcr-3*-negative strains for both drugs (Table 1), *mcr-3*-positive strains exhibited markedly reduced sensitivity to colistin, as expected. We next asked whether *mcr-3* enhances *in vivo* persistence under antibiotic pressure. Mice were infected individually with either *mcr-3*-positive or -negative strains and treated with ampicillin or an equal volume of PBS (Fig 1A). While ampicillin significantly lowered total bacterial burden, *mcr-3*-positive *E. coli* consistently exhibited higher organ colonization levels, regardless of treatment (Fig 1B-1F). Given that co-infections frequently occur in natural settings, we further assessed the competitive fitness of *mcr-3*-positive strains during mixed infection. Mice were co-infected with equal inocula of *mcr-3*-positive and *mcr-3*-negative strains, followed by ampicillin treatment (Fig 1G). The relative abundance of each strain recovered from the liver and spleen was determined by PCR screening of colonies. Approximately 90% of the recovered bacteria were *mcr-3*-positive (Fig 1H), indicating that *mcr-3* confers a competitive survival advantage even without direct resistance to the antibiotics used.

**Table 1:** MICs (mg/L) of tested antibiotics against *mcr-3*-positive/negative *E. coli.*

Strain Antibiotics	ATCC 25922	pHSG299 *Escherichia coli*	pHSG299-*mcr-3* *Escherichia coli*
**Colistin**	1	1	32
**Gentamicin**	1	0.5	0.5
**Ampicillin**	0.5	4	4

### *mcr-3* enables *E. coli* to evade intracellular killing by macrophages

**Fig 2:**
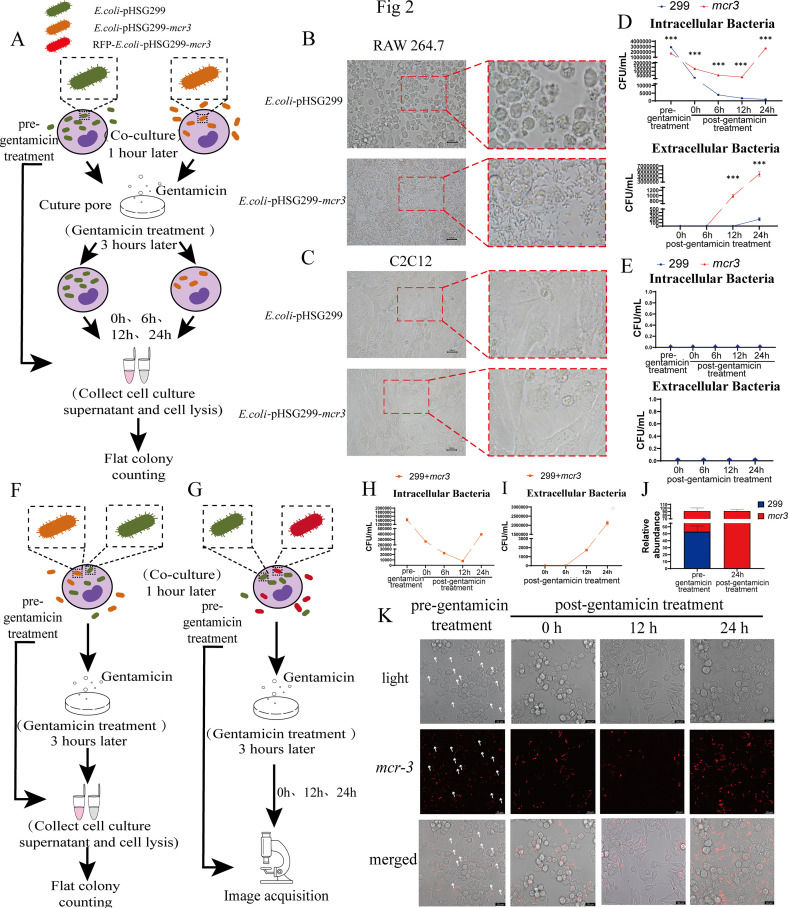
*mcr-3*-modified *E. coli* evade intracellular killing by macrophages. (A) Workflow of intracellular survival assay. RAW264.7 or C2C12 cells were infected with *mcr-3*-positive or -negative *E. coli* (MOI = 1:20) for 1 h; extracellular bacteria were eliminated by gentamicin (200 μg/mL, 3 h), and samples were collected at the indicated time points for CFU enumeration. (B) Phase-contrast microscopy of RAW264.7 cells at 24 h post-gentamicin treatment (scale bar, 100 μm). (C) Gentamicin efficacy control using non-phagocytic C2C12 cells, showing no extracellular bacteria at 24 h. (D-E) Intracellular and extracellular bacterial counts in RAW264.7 (D) and C2C12 (E) at indicated time points (n = 3 independent experiments, each in duplicate), “pre-gentamicin treatment” represents the 1-hour infection time point before gentamicin treatment, followed by measurements at 0, 6, 12, and 24 hours after gentamicin treatment. (F) Workflow of intracellular competition assay using a 1:1 mixture of *mcr-3*-positive and -negative *E. coli*. (G) Workflow of fluorescence-based competition assay using RFP-labeled *mcr-3*-positive and unlabeled negative strains. (H-I) Quantification of intracellular (H) and extracellular (I) bacteria following mixed infection (n = 3 independent experiments, each in duplicate). (J) Colony PCR identification of *mcr-3*-positive and -negative strains from 100 randomly picked extracellular colonies at 24 h post gentamicin treatment (n = 3 independent experiments). (K) Confocal microscopy of RAW264.7 infected with a 1:1 mixture of non-fluorescent *mcr-3*-negative and RFP-tagged *mcr-3*-positive *E. coli* at indicated time points. White arrows indicate *mcr-3*-negative bacteria lacking red fluorescence (scale bar, 20 μm). Data are shown as the mean ± SEM. Multiple t tests was conducted for pairwise comparisons. *P < 0.05, **P < 0.01, ***P ≤ 0.001; ns, not significant.

Macrophages are key effectors of innate immunity, responsible for engulfing and destroying invading bacteria. Our previous findings indicated that *mcr-3*-positive *E. coli* are taken up less efficiently by macrophages than *mcr*-negative bacteria, leading to more severe tissue damage and higher host mortality in infected animals. However, this alone did not fully explain the significantly higher survival of *mcr-3*-positive *E. coli* after antibiotic treatment. We therefore asked whether *mcr-3*-positive bacteria could survive and replicate within macrophages after phagocytosis (Fig 2A).

By 24 h post-gentamicin treatment, microscopy revealed numerous extracellular bacteria surrounding RAW264.7 macrophages infected with *mcr-3*-positive strains, whereas no extracellular bacteria were observed in cultures infected with *mcr-3*-negative strains (Fig 2B). To confirm that these extracellular bacteria resulted from macrophage release rather than from ineffective antibiotic killing, we performed parallel infections using the same bacterial strains in non-phagocytic C2C12 cells as a control. No bacteria were detected in C2C12 cultures either immediately or 24 hours after gentamicin treatment, confirming that gentamicin effectively eliminated extracellular bacteria (Fig 2C and 2E). After gentamicin treatment to remove extracellular bacteria, the intracellular *mcr-3*-negative bacteria were gradually eliminated, showing a decline in viable counts over time. In contrast, intracellular *mcr-3*-positive bacteria not only persisted but increased in number, indicating their survival and replication within macrophages (Fig 2D). Similar outcomes were observed using Bone Marrow-Derived Macrophages (BMDMs, S1 Fig).

To directly test competitive intracellular survival, we established an *in vitro* co-infection model (Fig 2F). Under 1:1 co-infection conditions, the overall intracellular and extracellular bacterial burden followed a trajectory similar to that observed for *mcr-3*-positive infection alone, showing a much higher intracellular load of bacteria at 24h than that at 12h (Fig 2H and 2I). To visualize the co-infection process and facilitate strain discrimination, macrophages were then co-incubated with *mcr-3*-positive *E. coli* expressing red fluorescent protein (RFP) and isogenic *mcr-3*-negative *E. coli* (non-fluorescent) at a 1:1 ratio (Fig 2G). At 24 h post-gentamicin treatment, abundant RFP-expressing bacteria were visible in the culture supernatant and around macrophages (Fig 2K), indicating sustained persistence of *mcr-3*-positive bacteria during co-infection, consistent with their competitive advantage within macrophages. To confirm the identity of surviving bacteria, a parallel co-infection assay was performed using the same experimental protocol. At 24 h post-gentamicin treatment, macrophages were lysed and the intracellular contents were plated on LB agar. From the resulting plates, 100 colonies were randomly picked and screened for the *mcr-3* gene by PCR: all colonies were *mcr-3* positive (Fig 2J). Together, these results demonstrate that *mcr-3* enables *E. coli* to survive macrophage-mediated killing and outcompete *mcr-3*-negative competitors within host cells.

### *mcr-3* reshapes macrophage immune and metabolic reprogramming induced by *E. coli*

**Fig 3:**
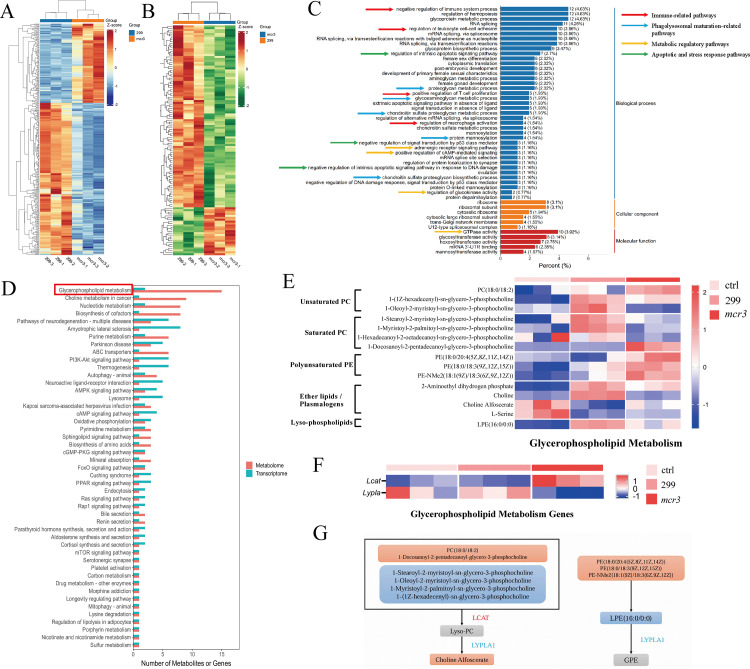
*mcr-3*-positive *E. coli* reprogram macrophage immune and metabolic pathways compared to *mcr-3*-negative *E. coli.* (A) Hierarchical clustering heatmap of differentially expressed genes (DEGs) in RAW264.7 cells infected with *mcr-3*-positive (*mcr3*) or -negative (299) *E. coli* (MOI = 1:20). After 1 h infection, cells were washed and treated with gentamicin to remove extracellular bacteria. Control cells received PBS and underwent the same washing and gentamicin treatment procedures. Cells were collected at 12 h post-gentamicin treatment (n = 3 independent experiments). (B) Hierarchical clustering heatmap of differentially expressed metabolites. (C) GO enrichment analysis of DEGs. (D) Integrated KEGG enrichment analysis of transcriptomic and metabolomic datasets. (E) Heatmap of differentially abundant metabolite enriched in glycerophospholipid metabolism. (F) Heatmap of DEGs associated with glycerophospholipid metabolism. (G) Reconstruction of glycerophospholipid metabolic pathway based on transcriptomic and metabolomic data. Orange boxes indicate upregulated metabolites in the *mcr-3* group, blue boxes indicate downregulated metabolites, and grey boxes indicate unchanged metabolites. Red and blue denote upregulated and downregulated genes, respectively. All metabolites within the black boxes are differentially altered phosphatidylcholines (PCs). Choline alfoscerate is a type of lysophosphatidylcholine (LPC). PE(18:0/20:4(5Z,8Z,11Z,14Z)), PE(18:0/18:3(9Z,12Z,15Z)), and PE-NMe2(18:1(9Z)/18:3(6Z,9Z,12Z)) are differentially altered phosphatidylethanolamines (PEs), and LPE(16:0/0:0) is a type of lysophosphatidylethanolamine (lyso-PE).

To elucidate the molecular mechanisms underlying the intracellular persistence of *mcr-3*-positive *E. coli*, we performed transcriptomic profiling of macrophages 12 hours post-gentamicin treatment with either *mcr-3*-positive or -negative strains. We identified 339 differentially expressed genes (DEGs), including 116 upregulated and 223 downregulated genes in the *mcr-3*-positive group relative to the *mcr-3*-negative group (Figs 3A and S2A). Complementary metabolomic analysis revealed 120 differential metabolites between macrophages infected with *mcr-3*-positive versus *mcr-3*-negative *E. coli*, including 25 upregulated and 95 downregulated in the *mcr-3*-positive group (Figs 3B and S2B).

Gene Ontology (GO) enrichment analysis of the DEGs showed predominant effects on immune system processes, lysosome maturation, metabolism, and stress response (Fig 3C). Notably, macrophages infected with *mcr-3*-positive *E. coli* exhibited significant downregulation of key immune-related processes such as leukocyte activation, response to cytokine stimulus, and overall immune system process. In parallel, pathways associated with phagolysosomal maturation, including lysosomal transport and endocytic vesicle maturation, were markedly downregulated in the *mcr-3*-positive group. These data suggest that *mcr-3*-positive bacteria induce functional alterations in macrophages, dampening immune activation and interfering phagosome maturation compared with *mcr-3*-negative bacteria.

Upon bacterial infection, macrophages undergo metabolic reprogramming characterized by shifts in glycolysis, oxidative phosphorylation, lipid metabolism, amino acid utilization, and iron handling—changes crucial for mounting effective antimicrobial responses [19]. Conversely, certain pathogens evade killing by re-wiring host metabolism, including glycolysis, mitochondrial function, lipid and amino acid metabolism, and iron homeostasis. These alterations result in reduced ROS production, impaired phagosome maturation, attenuated nitric oxide generation, and weaker iron-dependent bactericidal activity, collectively weakening macrophage antimicrobial functions and promoting intracellular bacterial survival [16,20,21]. We therefore integrated our transcriptomic and metabolomic data to identify coordinated metabolic changes in macrophages infected with *mcr-3*-positive *E. coli* (Fig 3D). This analysis revealed significant enrichment of pathways related to cellular energy metabolism (oxidative phosphorylation, AMPK, mTOR signaling) and lipid metabolism (glycerophospholipid and sphingolipid pathways). These changes likely influence membrane integrity and oxidative stress responses. We also observed alterations in amino acid and nucleotide metabolism, such as cysteine/methionine pathways, associated with immune activation and antioxidant defenses [16]. In addition, enrichment of porphyrin and nicotinamide metabolism suggested changes in iron handling and redox balance, implicating ferroptosis-related processes [21]. Several signal transduction pathways including PI3K-Akt, FoxO, and cAMP/cGMP-PKG signaling were also modulated, highlighting a broad integration of metabolic remodeling with immune signaling. Collectively, *mcr-3*-positive infection induces a comprehensive reprogramming of macrophage metabolism and immune function.

Glycerophospholipid metabolism was the most prominently altered pathway in the metabolomic dataset (Figs 3D and S2C), prompting focused analysis of its structural consequences. We observed a pronounced remodeling of phosphatidylcholine (PC) acyl-chain composition rather than uniform shifts across individual species (Fig 3E). Notably, PC(18:0/18:2)—a linoleoyl-containing unsaturated PC strongly associated with reduced bilayer order [22]—was selectively increased, while several saturated PCs, including 1-stearoyl-2-myristoyl-PC and 1-myristoyl-2-palmitoyl-PC, declined. The composite pattern of decreased saturated species together with the gain of a key unsaturated PC species indicates a broader disturbance of acyl-chain order, a physicochemical signature known to perturb membrane packing and compromise the stability of lipid-dependent protein assemblies [23]. Consistent with this membrane remodeling, we also observed perturbations in mitochondrial phospholipids, including accumulation of polyunsaturated phosphatidylethanolamine (PE) species such as PE(18:0/18:3) and PE(18:0/20:4), depletion of the PE head-group precursor phosphoethanolamine, and increased levels of the methylated intermediate PE-NMe₂(18:1/18:3). These signatures reflect impaired maintenance of mitochondrial inner-membrane architecture and coincide with altered mitochondrial ROS dynamics [24,25].

Alterations in upstream precursors, including choline, choline alfoscerate and L-serine, indicated increased flux through glycerophospholipid biosynthetic pathways. The shift in LPE(16:0) abundance further marked disruption of the deacylation-reacylation cycle, consistent with its role as a core intermediate in phospholipid remodeling [26]. Integration with transcriptomic data revealed upregulation of *Lcat* (Figs 3F and 3G and S3), a lecithin-cholesterol acyltransferase that converts PC to lysophosphatidylcholine [27], and downregulation of *Lypla1*, a cytosolic lysophospholipase that hydrolyzes lyso-PC and lyso-PE [28]. Although these enzymes act in distinct compartments, their transcriptional shifts parallel the depletion of saturated PCs and altered lyso-phospholipid pools, likely reflecting compensatory adjustments to disrupted phospholipid homeostasis. Representative MS/MS spectra of characteristic glycerophospholipid-related metabolites further supported that the major altered phospholipid signals were predominantly host-associated rather than simply reflecting increased bacterial lipid abundance (S4 Fig). Collectively, these findings demonstrate that *mcr-3*-positive *E. coli* elicit a glycerophospholipid-centric reprogramming of macrophage metabolism, disrupting membrane and mitochondrial integrity, weakening bactericidal function (e.g., ROS production, phagolysosome maturation), and broadly reshaping immune signaling to promote intracellular persistence.

### *mcr-3* reduces *E. coli*-induced ROS production in macrophages

During phagocytosis, activation of the NADPH oxidase complex on phagosomal membranes generates ROS, which are essential for oxidative bacterial killing [29]. In response, some bacteria counteract this defense by enhancing their antioxidant systems or actively suppressing host ROS production [16,30]. We therefore investigated whether *mcr-3* affects bacterial or macrophage oxidative stress responses.

**Fig 4:**
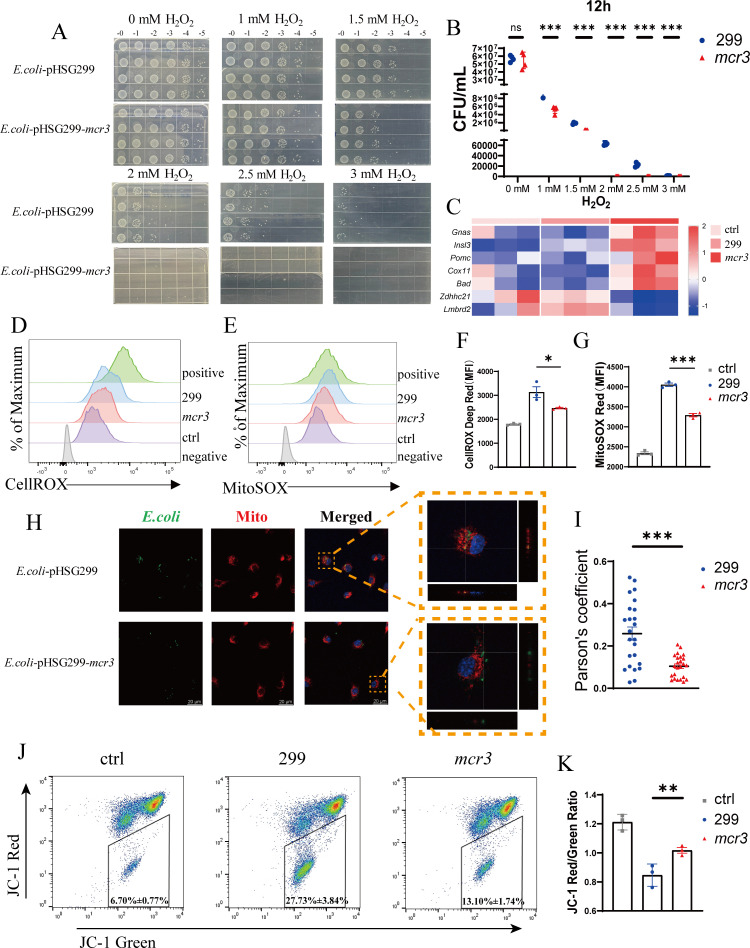
*mcr-3*-modified *E. coli* attenuate ROS production in macrophages. (A-B) Growth of *E. coli*-pHSG299 (299) and *E. coli*-pHSG299-*mcr-3* (*mcr3*) in LB medium containing 0–3 mM H_2_O_2_ for 12 h. Bacterial cultures were adjusted to an initial McFarland standard of 0.5, diluted 1:100, and incubated in the indicated H_2_O_2_ concentrations; bacterial viability was determined by serial dilution plating (n = 4 independent experiments). (C) Heatmap of DEGs related to metabolic regulation in infected macrophages (n = 3 independent experiments). (D-E) Representative flow cytometry analysis of total intracellular ROS and mitochondrial ROS in RAW264.7 cells infected with 299 or *mcr-3* for 1 h, assessed by CellROX and MitoSOX staining, respectively. H_2_O_2_-treated cells were included as a positive control. (F-G) Quantification of total intracellular ROS and mitochondrial ROS under the same conditions (n = 3 independent experiments). (H-I) Confocal microscopy of bacteria (green) and mitochondria (red) at 6 h post-infection. Colocalization was quantified by Pearson’s correlation coefficient (n = 24 cells per group from three independent experiments). Scale bar, 20 μm. (J-K) Mitochondrial membrane potential in RAW264.7 cells infected with 299 or *mcr-3* (MOI = 20:1). After 1 h infection, extracellular bacteria were removed by gentamicin, and JC-1 staining was performed at 12 h (n = 3 independent experiments). Data are shown as the mean ± SEM. Multiple t tests was conducted for pairwise comparisons. *P < 0.05, **P < 0.01, ***P ≤ 0.001; ns, not significant.

First, we compared the oxidative stress tolerance of *mcr-3*-positive and -negative bacteria by measuring growth in LB medium with increasing hydrogen peroxide (H₂O₂). At 2-, 2.5-, and 3-mM H₂O₂, *mcr-3*-positive strain showed significantly lower viability than the *mcr-3*-negative strain, indicating lower oxidative stress tolerance (Fig 4A and 4B). This suggests that *mcr-3* does not confer an advantage through enhanced antioxidant defense.

Next, transcriptomic profiling of macrophages infected with *mcr-3*-positive *E. coli* revealed marked changes in genes regulating energy metabolism and redox balance. Notably, *Gnas* and *Pomc* (activation of cAMP/PKA signaling), *Insl3* (immunosuppressive polarization), *Cox11* and *Bad* (stabilized mitochondrial electron transport) were upregulated [31–35], while *Zdhhc21* and *Lmbrd2* (modulation of membrane signaling) were downregulated [36–38] (Fig 4C). Together, they point to enhanced cAMP/PKA signaling and mitochondrial electron flow—both associated with reduced oxidative activity. Based on this transcriptional profile, we hypothesized that *mcr-3*-positive bacteria impair ROS generation in macrophages. We therefore quantified total and mitochondrial ROS following bacterial stimulation.

Consistently, functional assays showed that infection with *mcr-3*-positive *E. coli* significantly suppressed both total and mitochondrial ROS (mROS) production in macrophages compared to *mcr-3*-negative infection (Fig 4D-4G). Previous studies have shown that mROS are critical for macrophage bactericidal activity, with their production relying on mitochondrial recruitment to phagosomes and a corresponding reduction in mitochondrial membrane potential [39,40]. In line with this, *mcr-3*-positive *E. coli* significantly inhibited mitochondrial recruitment to phagosomes (Fig 4H and 4I) and attenuated the infection-induced reduction in mitochondrial membrane potential (Fig 4J and 4K).

### *mcr-3*-positive *E. coli* impairs phagolysosomal maturation

Lysosomes provide a hostile degradative environment for engulfed microbes; they contain acidic hydrolases and enzymes that help eliminate pathogens following phagosome-lysosome fusion [41]. Many bacteria, however, have evolved strategies to avoid destruction in phagolysosomes, either by tolerating acidic, enzyme-rich conditions or by actively interfering with phagosome maturation [41,42]. We examined whether *mcr-3* affects the ability of bacteria to cope with phagolysosomal conditions.

**Fig 5:**
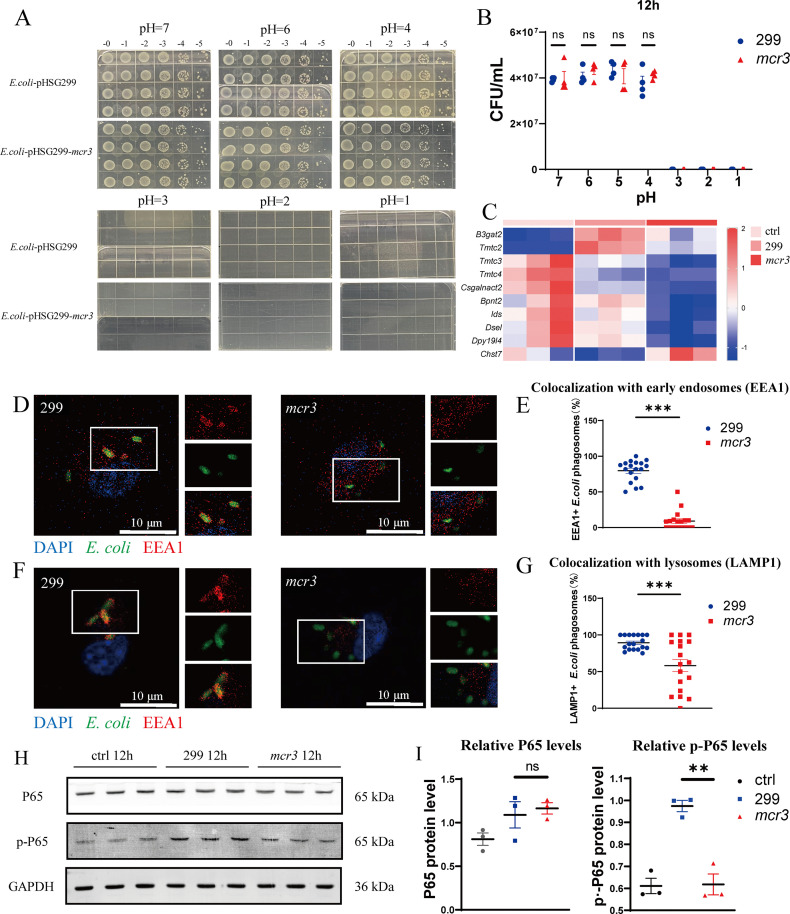
*mcr-3*-modified *E. coli* impair phagolysosomal maturation. (A–B) Growth of 299 and *mcr-3* strains in LB adjusted to pH 1–7 for 12 h. Cultures were adjusted to a McFarland standard of 0.5, diluted 1:100, and incubated at the indicated pH; bacterial viability was determined by serial dilution plating (n = 4 independent experiments). (C) Heatmap of DEGs related to phagolysosomal maturation in infected macrophages (n = 3 independent experiments). (D-G) RAW264.7 cells infected with 299 or *mcr-3* (MOI = 1:20, 6 h) were analyzed by confocal microscopy for colocalization of bacteria with EEA1 (early endosome marker) or LAMP1 (lysosome marker) (n = 18 cells per group from three independent experiments). Scale bar, 10 μm (H-I) Western blot analysis of P65 and p-P65 expression in RAW264.7 cells infected with 299 or *mcr-3* at an MOI of 1:20 for 1 h; extracellular bacteria removed by gentamicin, and cell lysates were collected at 12h post-gentamicin treatment for immunoblotting, with GAPDH used as the loading control (n = 3 independent experiments). Data are shown as the mean ± SEM. Multiple t tests was conducted for pairwise comparisons. *P < 0.05, **P < 0.01, ***P ≤ 0.001; ns, not significant.

First, we tested if *mcr-3*-positive *E. coli* displays enhanced ability to withstand an acidic environment, as has been reported for some intracellular pathogens like Staphylococcus aureus [43]. We adjusted the pH of the bacterial culture medium to acidic levels and compared growth of *mcr-3*-positive and *mcr-3*-negative strains. However, no significant difference in survival was observed between the two strains (Fig 5A and 5B), suggesting that acid tolerance is not a major factor in the intracellular survival advantage of *mcr-3*-positive strains.

Transcriptomic profiling revealed significant downregulation of genes involved in glycosaminoglycan and proteoglycan metabolism, including those annotated in “chondroitin sulfate proteoglycan biosynthetic process” and “protein mannosylation” pathways (Fig 3C). These pathways regulate glycosylation of phagosomal and lysosomal membrane proteins, which are essential for vesicle trafficking and phagolysosome maturation [44,45]. Notably, upregulation of *Chst7*, alongside widespread downregulation of glycosaminoglycan metabolic enzymes, suggested remodeling of this pathway with potential consequences for endolysosomal dynamics [46–49] (Fig 5C). These transcriptional changes prompted us to test whether phagolysosome maturation is impaired during *mcr-3* infection. To test this, we examined the intracellular localization of *E. coli* relative to phagosomal maturation markers. Compared to *mcr-3*-negative bacteria, *mcr-3*-positive strains showed largely reduced colocalization with EEA1 (an early endosome marker) and LAMP1 (a late endosome/lysosome marker) (Fig 5D-5G), indicating an arrest or delay in normal phagosome progression to mature phagolysosomes.

Previous studies have demonstrated that NF-κB activation promotes phagolysosomal maturation and enhances macrophage bactericidal activity [50,51], whereas some pathogens subvert this pathway to prolong intracellular survival [52]. In our earlier work, we found that *mcr-3*-modified LPS attenuates NF-κB activation and nuclear translocation [14]. We therefore hypothesized that *mcr-3*-positive *E. coli* impairs phagolysosomal maturation by suppressing NF-κB signaling. Consistent with this, macrophages infected with *mcr-3*-positive strains exhibited reduced NF-κB activation (Fig 5H and 5I), supporting a mechanistic link between disrupted immune signaling and impaired phagolysosomal trafficking.

### *mcr-3* enhances bacterial resistance to ferrous iron stress

**Fig 6:**
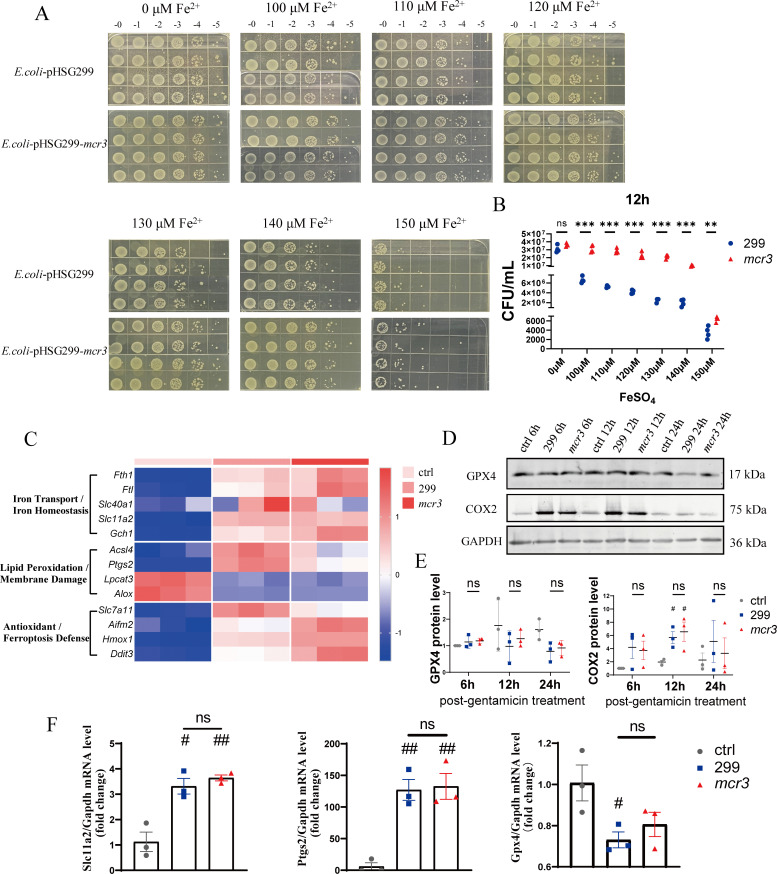
*mcr-3*–modified *E. coli* exhibit increased tolerance to ferrous ions. (A–B) Growth of 299 and *mcr-3* strains in LB supplemented with Fe^2+^ (0–150 μM) for 12 h. Cultures were adjusted to a McFarland standard of 0.5, diluted 1:100, and incubated at the indicated Fe^2+^ concentrations; bacterial viability was determined by serial dilution plating (n = 4 independent experiments). (C) Heatmap of DEGs related to ferroptosis pathways in infected macrophages (n = 3 independent experiments). (D-E) Western blot analysis of GPX4 and COX2 expression in RAW264.7 cells infected with 299 or *mcr-3* at an MOI of 1:20 for 1 h; extracellular bacteria removed by gentamicin, and cell lysates were collected at 6h, 12h, 24h post-gentamicin treatment for immunoblotting, with GAPDH used as the loading control (n = 3 independent experiments). (F) Relative mRNA levels of *Ptgs2, Slc11a2*, and *Gpx4* in RAW264.7 cells 12 h post-gentamicin treatment (n = 3 independent experiments). Data are shown as the mean ± SEM. Multiple t tests was conducted for pairwise comparisons. *P < 0.05, **P < 0.01, ***P ≤ 0.001; ns, not significant. ^#^ *P* < 0.05, ^##^ *P* < 0.01, ^###^ *P* ≤ 0.001 vs. control group.

Ferroptosis is an iron-dependent form of cell death driven by lipid peroxidation, characterized by oxidative damage to polyunsaturated fatty acids (PUFAs) in cellular membranes [53]. Recent studies have shown that macrophages can deploy a ferroptosis-like antimicrobial mechanism by transporting ferrous iron (Fe^2+^) into phagosomes via ferroportin, thereby catalyzing lethal lipid peroxidation in engulfed microbes [17]. In response, some bacteria counteract this process by producing high-affinity siderophores or other iron-sequestering factors that limit intraphagosomal iron availability and suppress ferroptotic killing [54]. To determine whether *mcr-3*-positive bacteria exhibit enhanced resistance to ferrous ions, we compared the growth of *mcr-3*-positive and -negative *E. coli* in media containing increasing concentrations of Fe^2+^ [17]. When Fe^2+^ levels exceeded 100 μM, *mcr-3*-positive *E. coli* showed significantly higher viability than their *mcr-3*-negative counterparts (Fig 6A and 6B), indicating improved tolerance to iron-mediated stress. To further compare the effects of different *mcr* genes on Fe^2+^ tolerance, we examined strains carrying *mcr-1*, *mcr-3*, or *mcr-9*. *mcr-1* increased bacterial viability under Fe^2+^ stress, whereas *mcr-9* did not show a comparable effect (S6A and S6B Fig). Notably, *mcr-3* expression conferred stronger Fe^2+^ tolerance overall than *mcr-1* (S6C and S6D Fig).

Although the transcriptomic analysis did not reveal differential expression of ferroptosis-related genes, particularly those involved in ferrous iron transport (Fig 6C), the observed oxidative and metabolic stress prompted us to examine ferroptosis activity in infected macrophages. We found no significant differences in ferroptosis levels between macrophages infected with *mcr-3*-positive versus -negative bacteria (Fig 6D-6F), likely due to comparable Fe^2+^ transport capacity in macrophages. These findings suggest that the differential intracellular survival of *mcr-3*-positive bacteria is unlikely to result from suppression of macrophage ferroptosis mediated by iron transport, but rather reflects their enhanced tolerance to ferrous iron.

## Discussion

Despite efforts to restrict polymyxin use in agriculture, *mcr-3*-bearing *E. coli* have persisted and even increased in prevalence in both clinical and livestock settings [4,9,55]. In China, *mcr-3* detections rose steadily from 2016-2019 even as *mcr-1* declined [9], suggesting that forces beyond direct colistin selection drive the success of *mcr-3* strains. One known factor is that, unlike MCR-1, the MCR-3 enzyme imposes minimal fitness cost on bacteria, avoiding the growth penalties associated with membrane perturbation [9]. However, the continued dominance of *mcr-3*-positive strains implies additional advantages during infection that extend beyond classical antibiotic resistance. Because bacterial fitness *in vivo* depends not only on resistance traits, but also on the ability to endure environmental stress [56] and evade host immunity [13], we hypothesized that *mcr-3* might enhance bacterial survival by subverting macrophage defenses, thereby linking antibiotic resistance to immune evasion.

To test this, we compared the infection outcomes of isogenic *mcr-3*-positive and -negative *E. coli*. As expected, *mcr-3* did not confer cross-resistance to other antibiotics *in vitro* — the minimum inhibitory concentrations of ampicillin and gentamicin were equivalent between *mcr-3*-harboring and control strains (Table 1). Nonetheless, under antibiotic treatment in mice, *mcr-3*-positive bacteria showed a clear survival advantage. In a mono-infection model, *mcr-3*-positive strains attained significantly higher tissue burdens than *mcr-3*-negative counterparts during ampicillin therapy (Fig 1B-1F). In competitive co-infections, the *mcr-3*-positive strain completely overran the *mcr-3*-negative strain in the presence of antibiotics (Fig 1G). These results indicate that even without higher intrinsic drug resistance, *mcr-3* allows pathogens to evade clearance under antibiotic pressure and exploit the host environment perturbed by treatment.

Macrophages emerged as a likely mediator of this advantage, given their central role in bacterial clearance. We observed that *mcr-3*-positive *E. coli* can actively subvert macrophage defenses to establish intracellular footholds. Previous reports noted that *mcr-3*-positive strains caused more severe disease in mice, possibly by avoiding phagocytosis [14]. Our findings extend this concept: in macrophage infection assays, *mcr-3*-positive bacteria survived and replicated within macrophages, whereas isogenic *mcr-3*-negative bacteria were efficiently killed (Figs 2B and 2D and S1). In mixed infections, virtually all intracellular bacteria recovered after 24 hours were positive for *mcr-3* (Fig 2F-2K). These data suggest that macrophages, instead of eradicating *mcr-3*-carrying bacteria, may unwittingly shelter them from antibiotics. By resisting phagocytic killing, *mcr-3*-harboring *E. coli* create protected intracellular reservoirs that allow them to persist and re-emerge once antibiotic levels wane.

To elucidate how *mcr-3*-positive *E. coli* reprograms macrophage behavior, we performed integrated transcriptomic and metabolomic analyses of infected cells. *mcr-3*-positive strains induced broad immunometabolic dysregulation by 12 hours post-gentamicin treatment (Fig 3C). Key pro-inflammatory and microbicidal genes in macrophages were markedly downregulated (S5A Fig), indicating a blunted inflammatory response [57–59]. Notably, pathways centered on glycerophospholipid metabolism were heavily perturbed (Figs 3D-3G, and S2C and S5B and S5C). We found an abnormal accumulation of phosphatidylcholine and phosphatidylethanolamine accompanied by depletion of their metabolic intermediates, pointing to impaired phospholipid turnover and membrane renewal. These results should be interpreted with caution. Because *mcr-3*-positive bacteria exhibited higher intracellular survival (Fig 2), there is a possibility that this apparent phospholipid remodeling partly reflects phospholipids derived from *E. coli* showing a more pronounced presence in the bulk metabolome. However, when the lipid annotations were considered together with the available MS/MS spectra (S4 Fig), the glycerophospholipid-related changes were mainly represented by PC/phosphocholine-containing lipids and PE species with polyunsaturated acyl chains. This differs from the typical *E. coli* membrane lipidome, which is dominated by PE, phosphatidylglycerol (PG), and cardiolipin and is characterized mainly by saturated, monounsaturated, or cyclopropanated acyl chains [60] Thus, although a minor bacterial contribution cannot be fully excluded, these changes more likely reflect macrophage-associated phospholipid remodeling. We further interpreted these metabolomic alterations together with the transcriptomic data as supporting evidence for host lipid metabolic changes. In parallel with this lipid remodeling, cellular energy balance was skewed: despite unchanged ATP levels, intracellular AMP and ADP were depleted (S5D and S5E Fig), suggesting inactivation of AMPK — a key regulator of autophagy, mitochondrial homeostasis, and ROS production [61]. In contrast, macrophages infected with the *mcr-3*-negative strain maintained dynamic phospholipid remodeling and normal choline utilization, hallmarks of an effective ongoing antimicrobial response [62–64]. Thus, *mcr-3*-positive *E. coli* drives a host metabolic shift focused on lipid and energy metabolism that is associated with reduced macrophage bactericidal function. This glycerophospholipid-centered reprogramming represents a cornerstone of how *mcr-3*-positive *E. coli* subverts host cell physiology.

A principal consequence of this immunometabolic rewiring is the suppression of ROS generation. *mcr-3*-positive *E. coli* infection created a cellular program that constrained oxidative burst from both NADPH oxidase and mitochondria. Transcriptomic data showed upregulation of Gαs-cAMP-PKA signaling components in infected macrophages (Fig 4C), which is known to inhibit NADPH oxidase activation and thereby dampen ROS production [34,35]. Meanwhile, expression of *Cox11*, which encodes a cytochrome c oxidase assembly factor, was increased; this likely stabilizes the electron transport chain and minimizes mROS leakage [32,33]. We also noted changes in additional regulators (e.g., *Bad*, *Zdhhc21*) consistent with maintaining mitochondrial integrity and reducing pro-oxidant signaling [36,37]. Together, these adaptations explain the dramatic drop in macrophage-derived ROS during *mcr-3*-positive *E. coli* infection. Another major outcome of *mcr-3*-positive *E. coli*-induced reprogramming is the blockade of phagolysosomal maturation. *mcr-3*-positive *E. coli* infection extensively disrupted genes involved in glycosaminoglycan and proteoglycan metabolism, crucial for endosomal trafficking and lysosomal function. We observed an imbalance in proteoglycan synthesis and turnover (e.g., *Chst7* up, *B3gat2*/*Csgalnact2* down) (Fig 5C), which likely impairs vesicle fusion events required for phagosome-lysosome fusion [47,48]. Thus, *mcr-3*-positive *E. coli* subverts macrophage antimicrobial capacity through a dual strategy: dampening oxidative killing and blocking phagolysosome development.

We validated these mechanistic insights through targeted functional assays. Macrophages infected with *mcr-3*-positive *E. coli* exhibited markedly reduced ROS output (Fig 4D-4G) and impaired phagosomal maturation (Fig 5D-5G), supporting the omics-based evidence of oxidative burst suppression and lysosomal blockade. Importantly, these functional impairments occurred despite a higher intracellular bacterial burden in the *mcr-3*-positive group, suggesting that increased bacterial-derived metabolites alone are unlikely to fully account for the observed host phenotypes, and are instead more consistent with a host-directed effect. Moreover, the bacteria themselves showed no increased tolerance to oxidative stress (Fig 4A and 4B) or acidity (Fig 5A and 5B), indicating that their survival advantage stems from host modulation rather than intrinsic resistance, a strategy increasingly recognized in clinically successful pathogens [43,65,66]. *mcr-3*-positive *E. coli* infection also disrupted mitochondrial dynamics, preventing normal trafficking to phagosomes and preserving mitochondrial membrane potential (Fig 4H-4K), thereby limiting mROS production [39,40]. In parallel, NF-κB activation was significantly reduced in macrophages infected with *mcr-3*-positive strains (Fig 5H and 5I) — consistent with prior reports using purified MCR-3-modified LPS [14]. Given the central role of NF-κB in inflammation and phagolysosomal maturation [50,51], this suppression likely contributes to both impaired antimicrobial signaling and vesicle trafficking. Together, these findings show that *mcr-3*-positive *E. coli* reprograms macrophage metabolism, signaling, and function to disable key antimicrobial defenses. By reshaping the intracellular environment, *mcr-3*-positive *E. coli* evade clearance and persist within host cells.

In addition to its host-directed effects, *mcr-3* also supports a bacteria-intrinsic adaptation that promotes intracellular persistence. Metabolomic profiling indicated that *mcr-3*-positive *E. coli* infection shifts macrophages toward a ferroptosis-prone state, reflected by altered lipid and redox metabolites [53]. However, no significant increase in ferroptosis was observed in infected macrophages (Fig 6C-6F), suggesting that host ferroptosis is not the primary driver of persistence. Instead, the key difference lay in bacterial tolerance to iron stress. *mcr-3*-positive *E. coli* displayed markedly higher survival under excess ferrous iron, while *mcr-3*-negative strains were more vulnerable to iron-mediated toxicity (Fig 6A and 6B). This implies that *mcr-3* enhances bacterial resistance to iron-dependent killing mechanisms deployed by macrophages. Notably, although Fe^2+^ supplementation in vitro does not fully recapitulate the ferroptosis-like environment within phagosomes, it isolates a key component of this process [17], namely iron-associated oxidative stress, which arises from the interaction between iron availability and local redox conditions. Accordingly, the improved survival of *mcr-3*-positive *E. coli* under Fe^2+^ exposure supports an increased capacity to withstand iron-dependent stress rather than direct modulation of host ferroptosis pathways. This form of stress extends beyond direct ROS-mediated damage and is further influenced by iron availability, redox cycling, and intracellular iron homeostasis [67]. Nonetheless, the precise bacterial mechanisms underlying this increased tolerance remain unclear and warrant further investigation. One possibility underlying this adaptation is that lipid A modification or associated envelope remodeling enhances iron sequestration or detoxification [67,68]. Consistent with this hypothesis, *mcr-1*, which modifies the same 4′-phosphate region of lipid A as *mcr-3*, also increased bacterial tolerance to Fe^2+^ stress, whereas *mcr-9*, which preferentially modifies the 1-phosphate region, did not show a comparable effect (S6A and S6B Fig). However, the *mcr-1*-mediated increase remained weaker overall than that conferred by *mcr-3* (S6C and S6D Fig). These results suggest that Fe^2+^ tolerance is unlikely to result from phosphoethanolamine addition alone. A plausible explanation is that lipid A phosphate groups participate in charge-dependent interactions with cationic molecules, including polymyxins and metal ions [69–71], and different patterns of phosphoethanolamine modification may alter these envelope-level interactions. This provides a plausible basis for the different Fe^2+^-tolerance phenotypes observed between *mcr-1*/*mcr-3*- and *mcr-9*-expressing strains, given their distinct lipid A modification-site preferences. However, the stronger effect of *mcr-3* than *mcr-1* indicates that modification position alone does not fully account for the magnitude of this phenotype. Previous studies have reported that site-selective lipid A phosphoethanolamine modification is linked to bacterial fitness [72], while *mcr-3* can impose a lower fitness burden than *mcr-1* in some experimental models [9,73]. Thus, differences in fitness cost may also influence the strength of Fe^2+^ tolerance, although the direct molecular basis remains to be clarified. Overall, the Fe^2+^-tolerance phenotype of *mcr-3*-positive strains, together with extensive host immune subversion, synergistically fortifies their intracellular survival.

**Fig 7:**
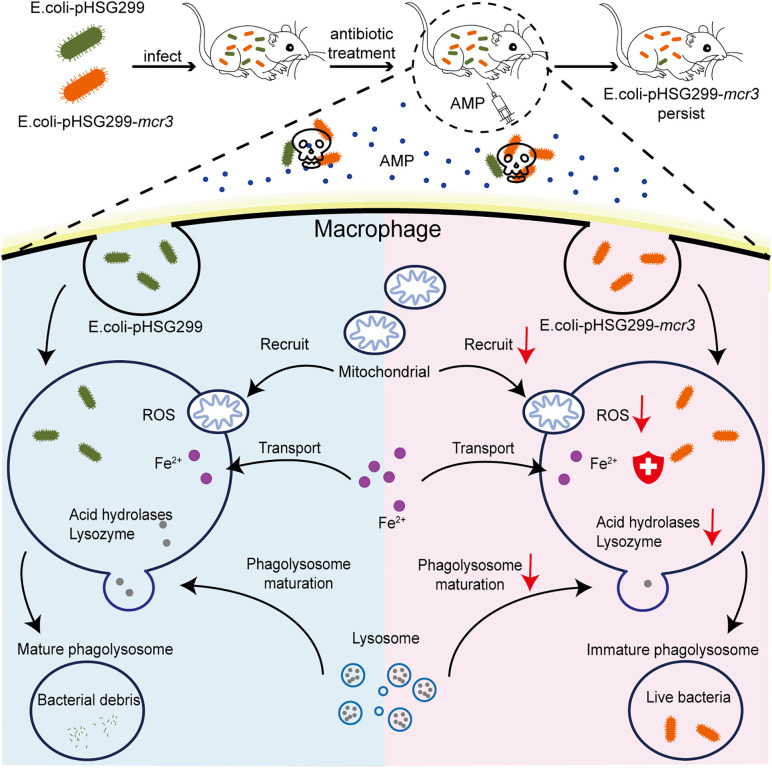
*mcr-3*-positive *E. coli* subvert macrophage defenses to establish intracellular niches and withstand antibiotic exposure. *mcr-3*-positive *E. coli* suppress ROS generation and block phagolysosomal maturation, while concurrently enhancing tolerance to ferrous iron and ferroptotic stress, enabling their persistence and competitive dominance *in vivo*.

In summary, our work reveals that the success of *mcr-3*-positive *E. coli* extends beyond antibiotic resistance. *mcr-3* functions as a dual-purpose virulence factor: it not only confers polymyxin resistance but also rewires host immunity to favor bacterial persistence. Mechanistically, *mcr-3*-positive strains suppress macrophage ROS production, block phagolysosomal maturation, and reprogram immunometabolic circuits, while concurrently enhancing tolerance to iron stress (Fig 7). Together, these host-directed and bacteria-intrinsic strategies establish an intracellular niche that shelters the pathogen during antibiotic exposure. This immune-evasive strategy represents a critical therapeutic challenge. Despite no *in vitro* differences in susceptibility, *mcr-3*-positive strains achieved clear competitive dominance *in vivo*, emphasizing how immune manipulation can drive treatment failure undetected by standard drug sensitivity tests. Of particular concern is the growing co-occurrence of *mcr-3* with other mcr genes (e.g., *mcr-1*) in single isolates [10]. Such convergence may foster highly persistent, multidrug-resistant lineages capable of evading both antibiotics and host defenses. Given the rising prevalence of *mcr-3*-positive and *mcr-1*/*mcr-3* co-harboring strains—even post-colistin bans—there is an urgent need to intensify surveillance of *mcr-3* dissemination and evolution. Future work should elucidate the molecular crosstalk between *mcr-3* and host immunity, which may uncover actionable targets to counteract the persistence and spread of these increasingly resilient pathogens.

## Materials and methods

### Ethics statement

All animal experiments were conducted in accordance with the Animal Management Rule of the Ministry of Health of the People’s Republic of China and the Guide for the Care and Use of Laboratory Animals published by the U.S. National Institutes of Health, and were approved by the Animal Care and Use Committee of China Agricultural University (approval number AW7121522-2-1).

### Mice

Six-week-old female BALB/c mice were purchased from Beijing Vital River Laboratory Animal Technology Co., Ltd. (Beijing, China). Mice were maintained under specific pathogen-free (SPF) conditions in the Laboratory Animal Center of China Agricultural University with ad libitum access to food and water. All infection experiments and tissue collection procedures were performed in an ABSL-2 facility.

### Murine infection model

For *in vivo* infection, bacteria were grown to logarithmic phase, washed, and resuspended in PBS. Mice were intraperitoneally injected with either *mcr-3*-positive or *mcr-3*-negative *E. coli* (5 × 10⁸ CFU/mL, 0.2 mL per mouse). Where indicated, mice received an intraperitoneal injection of ampicillin (320 mg/kg; Solarbio, Cat# IA0340) at 1 h post-infection. Control animals received equivalent volumes of PBS.

For competition assays, mice were injected with a 1:1 mixture of *mcr-3*-positive and -negative strains at the same total dose. Tissues were collected at 24 h post-infection for bacterial burden and strain ratio analyses.

### Bacterial strains and growth conditions

DH5α-pHSG299 and DH5α-pHSG299-*mcr-3* were constructed and stored in our laboratory [14]. ATCC 25922 was obtained from ATCC. DH5α-pHSG299-*mcr-1*, as well as the *mcr-1*- and *mcr-9*-expressing strains carrying pBAD-derived plasmids, together with the corresponding empty pBAD vector control strain, were provided by Wenjuan Yin and maintained in our laboratory. All *E. coli* strains were cultured with LB medium (LB Agar/Broth, LAND BRIDGE, Cat# CM159) containing Kanamycin (50 mg/L; Solarbio, Cat# K8020) at 37 °C. Strains carrying pBAD-derived plasmids were cultured in LB medium containing ampicillin (100 mg/L; Solarbio, Cat# IA0340) at 37 °C. ATCC 25922 was cultured in antibiotic-free LB medium at 37 °C.

### Cell lines and cell culture

RAW 264.7 and C2C12 cell lines were obtained from the Cell Resource Center at the Institute of Basic Medical Sciences, CAMS/PUMC (Beijing, China) and cultured with high-glucose DMEM-supplemented media (Gibco) contained with 10% fetal bovine serum (Gibco), 1% penicillin streptomycin combination (Solarbio) and 1% L-glutamine (Hyclone), plated onto 10 cm cell culture dishes.

For all experiments involving bacteria, including infection and gentamicin treatment to eliminate extracellular bacteria, cells were washed and cultured in antibiotic-free DMEM containing 1% fetal bovine serum and 1% L-glutamine. During gentamicin treatment, gentamicin (200 μg/mL) was added to the medium to remove extracellular bacteria [17].

### Bone marrow-derived macrophage isolation and culture

Bone marrow derived macrophages (BMDM) were isolated from 8-to-12-week-old mice. The femurs and tibias from mice were collected, and bone marrow cells were flushed with Hank’s (Hyclone) contained with 5% penicillin streptomycin combination, then cell suspensions were filtered with 100μm cell sterile strainer (Falcon) for removing cell clumps. Cell precipitates were collected by centrifugation and suspended in Hank’s. Cell suspensions treated with red blood cell lysis buffer (TIANGEN) for a few minutes and PBS (Hyclone) washed once. Finally, cell suspensions were suspended with RPMI 1640 Medium (Hyclone) contained with 20% fetal bovine serum, 1% penicillin streptomycin combination, 1% L-glutamine and 50 ng/mL MCSF (PeproTech, Cat# 315‑02), plated onto 10 cm cell culture dishes. After about three days, cells were digested using trypsin, centrifuged to obtain cell precipitates. Cell precipitates were suspended with RPMI 1640 Medium contained with 10% fetal bovine serum, 1% penicillin streptomycin combination, 1% L-glutamine and 50 ng/mL MCSF [74]. After about two days BMDMs could be obtained. BMDMs were treated with 100 ng/mL LPS (Sigma, Cat# L2880) for 6 hours to induce activation.

### Determination of bacterial burden and competitive index

Hearts, livers, spleens, lungs, and kidneys were aseptically collected from *E. coli*-infected mice. The tissues were weighed and homogenized in sterile PBS. The resulting homogenates were serially diluted and plated onto LB agar plates (LAND BRIDGE), followed by incubation at 37 °C for 18–24 hours. The total number of colony-forming units (CFUs) in each sample was counted, and the bacterial burden was calculated as CFU per gram of tissue.

To assess the *in vivo* competitive advantage of *mcr-3*-positive *E. coli*, spleen and liver tissues were collected from the same group of mice infected with a 1:1 mixture of *mcr-3*-positive and -negative strains. The tissues were homogenized in PBS and plated on LB agar. After incubation at 37 °C for 18–24 hours, 100 colonies were randomly selected from each plate and subjected to PCR analysis to determine the presence of the *mcr-3* gene. The proportion of *mcr-3*-positive colonies was then calculated to evaluate the relative fitness of the resistant strain under host selective pressure.

### MIC determination

To exclude potential bias caused by differential antibiotic susceptibility, the minimum inhibitory concentrations (MICs) of ampicillin and gentamicin were determined for both *E. coli* strains prior to their use in *in vivo* and *in vitro* experiments, respectively. The MICs against ATCC25922, DH5α-pHSG299 and DH5α-pHSG299-*mcr-3* were determined using a broth dilution method according to the Clinical and Laboratory Standards Institute document M45‐A2. In this experiment, ATCC 25922 served as the control strain. Bacteria in the logarithmic phase were adjusted to a turbidity equivalent to 0.5 McFarland standard, diluted 1:100, and inoculated into 96-well plates (Nantong Jiange Experimental Equipment Co., Ltd., China) containing serial twofold dilutions of colistin (Solarbio, Cat# IC0520; concentrations ranging from 0.5 to 64 mg/L), gentamicin (Solarbio, Cat# G8170; concentrations ranging from 0.0625 to 8 mg/L) or ampicillin (Solarbio, Cat# IA0340; concentrations ranging from 0.0625 to 8 mg/L)using CA-MHB (Hopebio). Plates were incubated at 37 °C for 16–20 hours, and bacterial growth was assessed visually to determine the lowest antibiotic concentration that completely inhibited visible growth.

### Microscopy

RAW264.7 cells were placed in 6-well plates or 35-mm glass-bottom confocal dishes (NEST, Cat# 801001). BMDMs and C2C12 cells were placed in 6-well plates.

RAW264.7 and C2C12 cells in 6-well plates were infected with *mcr-3*-positive/negative *E. coli* at an MOI of 1:20 for an hour. Then extracellular bacteria were removed by gentamicin treatment (200 μg/mL) for 3 hours. At 24 hours post-gentamicin treatment, these 6-well plates were visualized with a Nikon Eclipse Ti microscope (Nikon). In addition, BMDMs were treated following the same procedure, and samples were collected at 0 and 24 h after gentamicin treatment.

RAW264.7 cells in confocal dishes were cultured with a 1:1 mixture of red fluorescent proteins (RFP)-labeled *mcr-3*-positive *E. coli* and unlabeled *mcr-3*-negative *E. coli* at an MOI of 1:20 for an hour, then extracellular bacteria were removed by gentamicin treatment for 3 hours. Images were obtained by Leica TCS‐SP8 confocal microscope at 1 h post-infection (before gentamicin treatment) and 0, 12, 24 hours after gentamicin treatment.

### Quantification of intracellular and extracellular bacteria

RAW 264.7 cells were seeded into 24-well plates and infected with either *mcr-3*-positive, *mcr-3*-negative, or a 1:1 mixture of *mcr-3*-positive and negative *E. coli* strains at a multiplicity of infection (MOI) of 1:20 for 1 hour. After infection, extracellular bacteria were removed by gentamicin treatment (200 μg/mL) for 3 hours. At 1 h post-infection (before gentamicin treatment) and at 0, 6, 12, and 24 h after gentamicin treatment the culture supernatant was collected to assess extracellular bacterial load. The cells were then washed twice with PBS and lysed by adding 0.5 mL of 1% (v/v) Triton X-100 (Sigma, Cat# T8787) per well. Both the supernatants and cell lysates were serially diluted and plated onto LB agar plates to quantify extracellular and intracellular bacterial loads, respectively.

To assess the competitive advantage of *mcr-3*-positive strains specifically under co-infection conditions, bacterial colonies were randomly picked from the LB plates at 24 hours post-gentamicin treatment only in the mixed infection group. From each plate, 100 colonies were randomly selected and subjected to PCR analysis to detect the *mcr-3* gene. The proportion of *mcr-3*-positive colonies was then calculated for both extracellular and intracellular populations. The primers used in the experiments were *M13* 5’- CGCCAGGGTTTTCCCAGTCACGAC -3’ 5’- AAGCGGATAACAATTTCACACAGGA -3’.

### Immunofluorescence and confocal microscopy

BMDMs were placed in cell slides. BMDMs were infected with *mcr-3*-positive/negative *E. coli* at an MOI of 1:20 for 6 hours and fixed with paraformaldehyde. Then BMDMs were incubated with anti-*E. coli* (Abcam, Cat# 30522) and anti-EEA1 (Cell Signaling Technology, Cat# 48453) or anti-LAMP1 (Cell Signaling Technology, Cat# 15665) or HSP60 (Proteintech, Cat# 66041–1-Ig) antibodies [75]. As secondary antibodies, Alexa 594 and 488 (Cell Signaling Technology) were added for 1h at room temperature in dark, and then sections were sealed by fluorescent mounting medium with DAPI (ZSGB-BIO) in dark. These sections were visualized with Leica TCS‐SP8 confocal microscope. Images were analyzed with Leica Application Suite X software.

### Measurement of ROS and mROS

RAW 264.7 cells were placed in 12-well plates and infected with *mcr-3*-positive/negative *E. coli* at an MOI of 1:20 for an hour. For positive controls, RAW264.7 cells were treated with 600 μM H_2_O_2_ for 10 h before CellROX staining or with 800 μM H_2_O_2_ for 10 h before MitoSOX staining [76,77]. The culture medium was removed and then the cells were washed with PBS and then incubated for 30 min at 37 °C with CellROX (Invitrogen, Cat# C10422) or MitoSOX (Invitrogen, Cat# M36008) at a final concentration of 5 μM in serum-free DMEM (Invitrogen). The cells were washed with warmed PBS, removed from the plates by pipetting with pancreatin, pelleted at 1500 r.p.m. for 3 min, immediately resuspended in cold PBS containing 1% FBS and analyzed by flow cytometry (BD FACSAria III).

### Assessment of mitochondrial membrane potential

The mitochondrial membrane potential (ΔΨm) was assessed using a JC-1 mitochondrial membrane potential assay kit (Solarbio, Cat# M34152). RAW264.7 cells were seeded in 12-well plates and infected with *mcr-3*-positive or -negative *E. coli* at a multiplicity of infection (MOI) of 1:20 for 1 h. Extracellular bacteria were subsequently removed by treatment with gentamicin for 3 h. Then cells were washed three times with PBS and further cultured in antibiotic-free DMEM supplemented with 1% FBS. At 12 h post-gentamicin treatment, cells were incubated with JC-1 working solution at 37 °C for 20 min in the dark. Then, the cells were washed with warmed PBS, removed from the plates by pipetting with pancreatin, pelleted at 1500 r.p.m. for 3 min, immediately resuspended in cold PBS containing 1% FBS and analyzed by flow cytometry. The green (JC-1 monomer) and red (JC-1 aggregate) fluorescence were detected with FITC (488 nm) and PE (585 nm) channels respectively. FlowJo software was used for quantitative analysis.

### Bacterial stress tolerance assays

To evaluate the intracellular stress adaptability of *mcr-3*-positive/negative *E. coli* strains, bacterial tolerance to oxidative, acidic, and ferrous conditions was assessed [17,78]. For oxidative stress assays, hydrogen peroxide (H₂O₂) was added to LB broth to final concentrations of 0, 1.0, 1.5, 2.0, 2.5, and 3.0 mM. For acid stress assays, the pH of LB broth was adjusted to 1, 2, 3, 4, 5, 6, or 7 using hydrochloric acid or sulfuric acid. For iron stress assays, ferrous sulfate was added to LB broth to final Fe^2+^ concentrations of 0, 100, 110, 120, 130, 140, and 150 μM. For the additional ferrous ion stress assays, the *mcr-1*- and *mcr-9*-expressing strains and the corresponding empty vector control were assessed under the same ferrous ion stress conditions.

All stress-conditioned media were sterilized using 0.22-μm filters (Millex, Cat# SLGPR33RS). *E. coli* strains in logarithmic growth phase were adjusted to a turbidity of 0.5 McFarland units, and then diluted 1:100 into the prepared LB broth containing various concentrations of H₂O₂, pH, or Fe^2+^ levels. The cultures were incubated at 37 °C for 12 hours. Bacterial survival and growth were determined by serial dilution followed by spot plating on LB agar.

### Immunoblotting

Total proteins were extracted from RAW264.7 cells following infection with *E. coli*-pHSG299 or *E. coli*-pHSG299-*mcr-3* at a multiplicity of infection (MOI) of 1:20 for 1 h. Extracellular bacteria were removed by gentamicin treatment (200 μg/mL), and cell lysates were collected at the indicated time points post-gentamicin treatment. Cells were lysed with RIPA lysis buffer (Solarbio). Proteins were separated by SDS-PAGE (GenStar), then transferred to PVDF membranes (MILLIPORE). PVDF membranes were incubated with the following primary antibodies overnight at 4 °C: anti-P65 (Selleck, Cat# 0155), anti-p-P65 (Selleck, Cat# 0006), anti-GPX4 (Abmart, Cat# 56959), anti-COX2 (Abmart, Cat# 58852), anti-GAPDH (ZCGB BIO, Cat# TA-08). DyLight 680 and 800-conjugated secondary antibodies (Cell Signaling Technology) incubated for 1h at room temperature. The membranes were analyzed by Azure Sapphire (Azure biosystems).

### Quantitative real-time PCR

RAW 264.7 cells infected with *mcr-3*-positive/negative *E. coli* at an MOI of 1:20 for an hour, extracellular bacteria were eliminated by gentamicin treatment for 3 hours. At 12 hours post-gentamicin treatment, RNA was extracted through the Trizol reagent method (Thermo Fisher). 1000ng RNA was reverse transcribed to cDNA using Thermo Scientific RevertAid RT (Thermo). Aliquots of the reaction mixture were used for qRT-PCR analysis. The primers used in the experiments were *Slc11a2* 5’- CTGATCGTCTGCTCCATCAA -3’ 5’- CCCAATGCAATCAAACACTG -3’, *Ptgs2* 5’- GAAATATCAGGTCATTGGTGGAGA -3’ 5’- ATGCTCCTGCTTGAGTATGTCG -3’, *Gpx4* 5’- GCTGGGAAATGCCATCAAAT -3’ 5’- TCCTTCTCTATCACCTGGGGCT -3’, *Gapdh* 5’-TGCCCCCATGTTTGTGATG-3’ 5’-TGTGGTCATGAGCCCTTCC-3’.

To assess the potential effect of gentamicin treatment, RAW264.7 cells were left uninfected and cultured in the presence or absence of gentamicin [ctrl (+GM) and ctrl (−GM), respectively]. After 3 h of incubation, cells were washed with PBS and incubated in antibiotic-free medium for an additional 12 h. Cells were then harvested, and total RNA was extracted for RT-qPCR analysis as described above (S7 Fig). The primers used in the experiments were *Lcat* 5’- CCACCAGCAGGATGAATACTACAAG -3’ 5’- GCTATGCCCAATGAGGAAGACAG -3’, *Lypla* 5’- GGAATTAAACAGGCAGCAGAAACC -3’ 5’- ATGGCACTGGAGAACGGAAATATC -3’, *Pcyt1a* 5’- GCAGGGAGCGATGATGTGTATAAG -3’ 5’- GTGATGATGTCTGATGTGGAGATACC -3’, *Gnas* 5’- TCTGTGGGAGGATGAGGGAG -3’ 5’- TGGTCACTTGGCACGTAGTC -3’, *Bad* 5’- AGAGTTTGAGCCGAGTGAGC -3’ 5’- CCGTCCCTGCTGATGAATGT -3’, *Zdhhc21* 5’- GCTGCATGGGCTTGATTGTC -3’ 5’- CCCTCACTAAGGCAACCAGG -3’.

Relative expression levels were calculated using the 2^-ΔCt method with GAPDH as an internal control.

### Transcriptomic and metabolomic analyses

RAW264.7 cells were infected with either *mcr-3*-positive or *mcr-3*-negative *E. coli* strains at a multiplicity of infection (MOI) of 1:20 for 1 hour. Control cells were left uninfected and subjected to the same subsequent procedures. After incubation, cells were washed three times with PBS, followed by incubation with DMEM containing 1% FBS and gentamicin (200 μg/mL) for 3 h to eliminate extracellular bacteria. After gentamicin treatment, cells were washed three times with PBS and further cultured in antibiotic-free DMEM supplemented with 1% FBS for an additional 12 h. Before sample collection, cells were washed with pre-cooled PBS and harvested for transcriptomic and metabolomic analyses. Total RNA was extracted using Trizol reagent for transcriptomic sequencing, while cells for metabolomic profiling were collected using pre-cooled PBS. All sample preparations, transcriptomic sequencing, and metabolomic analyses were performed by Wuhan MetWare Biotechnology Co., Ltd. (Wuhan, China). KEGG and GO enrichment analyses, as well as volcano plots, were generated by MetWare. Heatmaps were generated based on Z-score-normalized data using GraphPad Prism 8.

## Statistical analysis

All statistical analyses were performed using GraphPad Prism 8. Data are presented as mean ± SEM. Statistical tests, sample sizes, and exact *P* values are indicated in the figure legends. Schematic drawings presented in this paper were created with Adobe Illustrator (Adobe Illustrator 2020).

## Supporting information

S1 Fig Validation of intracellular persistence in primary macrophages. (A) Representative microscopy of LPS-activated BMDMs at 0 h and 24 h post-gentamicin treatment with 299 or *mcr-3* strains. Extracellular bacteria were detected only in the *mcr-3* group at 24 h. Scale bar, 100 μm. (B) Quantification of intracellular bacteria in BMDMs at indicated time points (n = 3 independent experiments, each in duplicate). Data are shown as the mean ± SEM. Multiple t tests was conducted for pairwise comparisons. *P < 0.05, **P < 0.01, ***P ≤ 0.001; ns, not significant.
(TIF)

S2 Fig Transcriptomic and metabolomic alterations in RAW264.7 macrophages. (A) Volcano plot of DEGs between *mcr-3* and 299 groups. Red and blue dots indicate significantly up- and downregulated genes. (B) Volcano plot of differential metabolites. Red and green dots indicate significantly up- and downregulated metabolites. (C) KEGG pathway enrichment of differential metabolites; y-axis, pathway names; x-axis, number and percentage of mapped metabolites.
(TIF)

S3 Fig Differential metabolites and genes in glycerophospholipid metabolism. Differential metabolites and genes were simultaneously mapped onto the glycerophospholipid metabolism pathway (KEGG map: ko00564). Circles and squares represent metabolites and genes, respectively. Red indicates upregulated metabolites or genes, blue indicates downregulated metabolites or genes, and yellow circles indicate metabolites with both up- and down-regulated states. Green squares highlight the two regions with the most concentrated changes. 2.3.1.43: *Lcat*; 3.1.1.5: *Lypla1*.
(TIF)

S4 Fig Representative MS/MS spectra of glycerophospholipid-related metabolites. (A-D) Representative MS/MS spectra of PC and phosphocholine-containing lipids acquired in positive-ion mode. (E) Representative MS/MS spectrum of PE(18:0/20:4) acquired in negative-ion mode. (F) Representative MS/MS spectrum of LPE(16:0/0:0) acquired in positive-ion mode. Major fragment ions are labeled. Red annotations indicate diagnostic fragment ions used to support metabolite annotation.
(TIF)

S5 Fig Heatmaps of GO- and KEGG-associated changes. (A) Heatmap of DEGs enriched in GO terms related to macrophage activation. (B-E) Heatmaps of metabolites enriched in KEGG pathways from metabolomic profiling.
(TIF)

S6 Fig Effects of *mcr* gene expression on bacterial tolerance to Fe^2+^ stress. (A-B) Growth of Vector, *mcr-1*, and *mcr-9* strains carrying pBAD-derived plasmids in LB supplemented with the indicated concentrations of FeSO_4_ for 12 h. (C-D) Growth of Vector, *mcr-1*, and *mcr-3* strains carrying pHSG299-derived plasmids in LB supplemented with the indicated concentrations of FeSO_4_ for 12 h. Cultures were adjusted to a McFarland standard of 0.5, diluted 1:100, and incubated at the indicated Fe^2+^ concentrations; bacterial viability was determined by serial dilution plating (n = 4 independent experiments). Data are shown as the mean ± SEM. Multiple t tests was conducted for pairwise comparisons. *P < 0.05, **P < 0.01, ***P ≤ 0.001 compared with the Vector control; ^*#*^*P* < 0.05, ^*##*^*P* < 0.01, ^*###*^*P* ≤ 0.001 for comparisons between *mcr-1* and *mcr-9* in A-B or between *mcr-1* and *mcr-3* in C-D; ns, not significant. For panel D, the overall Fe^2+^-tolerance profiles of *mcr-1*- and *mcr-3*-carrying strains were additionally compared by ordinary two-way ANOVA using log_10_-transformed CFU values, with the corresponding *P* values indicated above the graph.
(TIF)

S7 Fig qRT-PCR validation of selected genes to evaluate potential effects of gentamicin treatment. RAW264.7 cells were left uninfected and cultured either with or without gentamicin, defined as ctrl (+GM) and ctrl (−GM), respectively (n = 3 independent experiments). (A-C) Expression of glycerophospholipid metabolism-related genes (*Lcat*, *Lypla* and *Pcyt1a*). (D-F) Expression of representative differentially expressed genes identified from transcriptomic analysis (*Gnas*, *Bad*, and *Zdhhc21*). Gene expression levels were normalized to the internal control and are presented relative to the ctrl (+GM) group. Data are shown as the mean ± SEM. Multiple t tests was conducted for pairwise comparisons. ns, not significant.
(TIF)

S1 Data Source data underlying all quantitative results presented in the main and supplementary figures. Separate worksheets are provided for each figure and labeled according to the corresponding figure number.
(XLSX)

S1 File Raw images. **Original uncropped and unadjusted images underlying all blot results.** (A) Original images for P65, p-P65, and GAPDH blots corresponding to Fig 5H. (B) Original images for GPX4, COX2, and GAPDH blots corresponding to Fig 6D. Molecular weights of the detected proteins are indicated on the right. Where applicable, membranes were cut before antibody incubation or imaging, and the full captured image of each resulting membrane strip is shown.
(PDF)

## References

[1] Nadgir CA, Biswas DA. Antibiotic Resistance and Its Impact on Disease Management. Cureus. 2023;15(4):e38251. doi: 10.7759/cureus.38251 37261148 PMC10226836

[2] Paterson DL, Harris PNA. Colistin resistance: a major breach in our last line of defence. Lancet Infect Dis. 2016;16(2):132–3. doi: 10.1016/S1473-3099(15)00463-6 26603171

[3] Xu Y, Zhong L-L, Srinivas S, Sun J, Huang M, Paterson DL, et al. Spread of MCR-3 Colistin Resistance in China: An Epidemiological, Genomic and Mechanistic Study. EBioMedicine. 2018;34:139–57. doi: 10.1016/j.ebiom.2018.07.027 30061009 PMC6116419

[4] Ling Z, Yin W, Shen Z, Wang Y, Shen J, Walsh TR. Epidemiology of mobile colistin resistance genes mcr-1 to mcr-9. J Antimicrob Chemother. 2020;75(11):3087–95. doi: 10.1093/jac/dkaa205 32514524

[5] Liu Y-Y, Wang Y, Walsh TR, Yi L-X, Zhang R, Spencer J, et al. Emergence of plasmid-mediated colistin resistance mechanism MCR-1 in animals and human beings in China: a microbiological and molecular biological study. Lancet Infect Dis. 2016;16(2):161–8. doi: 10.1016/S1473-3099(15)00424-7 26603172

[6] Wang C, Feng Y, Liu L, Wei L, Kang M, Zong Z. Identification of novel mobile colistin resistance gene mcr-10. Emerg Microbes Infect. 2020;9(1):508–16. doi: 10.1080/22221751.2020.1732231 32116151 PMC7067168

[7] Liu M, Wu J, Zhao J, Xi Y, Jin Y, Yang H, et al. Global epidemiology and genetic diversity of mcr-positive Klebsiella pneumoniae: A systematic review and genomic analysis. Environ Res. 2024;259:119516. doi: 10.1016/j.envres.2024.119516 38950813

[8] Rhouma M, Madec J-Y, Laxminarayan R. Colistin: from the shadows to a One Health approach for addressing antimicrobial resistance. Int J Antimicrob Agents. 2023;61(2):106713. doi: 10.1016/j.ijantimicag.2023.106713 36640846

[9] Liang L, Li Y, Wang L, Wang W, Zhang Y, Zhao H, et al. Fitness costs of mobilised colistin resistance gene 3 (mcr-3): systematic review, epidemiological study, and functional analysis. EBioMedicine. 2025;120:105923. doi: 10.1016/j.ebiom.2025.105923 40945051 PMC12571581

[10] Li J, Chang J, Ma J, Zhou W, Yang Y, Wu J, et al. Genome-based assessment of antimicrobial resistance of Escherichia coli recovered from diseased swine in eastern China for a 12-year period. mBio. 2025;16(5):e0065125. doi: 10.1128/mbio.00651-25 40243369 PMC12077178

[11] Stapels DAC, Hill PWS, Westermann AJ, Fisher RA, Thurston TL, Saliba A-E, et al. Salmonella persisters undermine host immune defenses during antibiotic treatment. Science. 2018;362(6419):1156–60. doi: 10.1126/science.aat7148 30523110

[12] Wheatley R, Diaz Caballero J, Kapel N, de Winter FHR, Jangir P, Quinn A, et al. Rapid evolution and host immunity drive the rise and fall of carbapenem resistance during an acute Pseudomonas aeruginosa infection. Nat Commun. 2021;12(1):2460. doi: 10.1038/s41467-021-22814-9 33911082 PMC8080559

[13] PLOS Pathogens Staff. Correction: Bacterial immune evasion through manipulation of host inhibitory immune signaling. PLoS Pathog. 2015;11(4):e1004813. doi: 10.1371/journal.ppat.1004813 25853824 PMC4390327

[14] Yin W, Ling Z, Dong Y, Qiao L, Shen Y, Liu Z. Mobile colistin resistance enzyme MCR-3 facilitates bacterial evasion of host phagocytosis. Adv Sci. 2021;8:e2101336. doi: 10.1002/advs.202101336 PMC8456205 34323389

[15] Schator D, G Kumar N, Chong SJU, Jung TK, Jedel E, Smith BE, et al. Cross-membrane cooperation among bacteria can facilitate intracellular pathogenesis. Nat Commun. 2025;16(1):7419. doi: 10.1038/s41467-025-62575-3 40790115 PMC12339937

[16] Leseigneur C, Lê-Bury P, Pizarro-Cerdá J, Dussurget O. Emerging Evasion Mechanisms of Macrophage Defenses by Pathogenic Bacteria. Front Cell Infect Microbiol. 2020;10:577559. doi: 10.3389/fcimb.2020.577559 33102257 PMC7545029

[17] Ma R, Fang L, Chen L, Wang X, Jiang J, Gao L. Ferroptotic stress promotes macrophages against intracellular bacteria. Theranostics. 2022;12(5):2266–89. doi: 10.7150/thno.66663 35265210 PMC8899587

[18] Huang L, Nazarova EV, Russell DG. Mycobacterium tuberculosis: Bacterial Fitness within the Host Macrophage. Microbiol Spectr. 2019;7(2):10.1128/microbiolspec.bai-0001–2019. doi: 10.1128/microbiolspec.BAI-0001-2019 30848232 PMC6459685

[19] Sweet MJ, Ramnath D, Singhal A, Kapetanovic R. Inducible antibacterial responses in macrophages. Nat Rev Immunol. 2025;25(2):92–107. doi: 10.1038/s41577-024-01080-y 39294278

[20] Cumming BM, Addicott KW, Adamson JH, Steyn AJ. Mycobacterium tuberculosis induces decelerated bioenergetic metabolism in human macrophages. Elife. 2018;7:e39169. doi: 10.7554/eLife.39169 30444490 PMC6286123

[21] Nairz M, Schroll A, Sonnweber T, Weiss G. The struggle for iron - a metal at the host-pathogen interface. Cell Microbiol. 2010;12(12):1691–702. doi: 10.1111/j.1462-5822.2010.01529.x 20964797

[22] Kanno K, Wu MK, Scapa EF, Roderick SL, Cohen DE. Structure and function of phosphatidylcholine transfer protein (PC-TP)/StarD2. Biochim Biophys Acta. 2007;1771(6):654–62. doi: 10.1016/j.bbalip.2007.04.003 17499021 PMC2743068

[23] Casares D, Escribá PV, Rosselló CA. Membrane Lipid Composition: Effect on Membrane and Organelle Structure, Function and Compartmentalization and Therapeutic Avenues. Int J Mol Sci. 2019;20(9):2167. doi: 10.3390/ijms20092167 31052427 PMC6540057

[24] Tasseva G, Bai HD, Davidescu M, Haromy A, Michelakis E, Vance JE. Phosphatidylethanolamine deficiency in Mammalian mitochondria impairs oxidative phosphorylation and alters mitochondrial morphology. J Biol Chem. 2013;288(6):4158–73. doi: 10.1074/jbc.M112.434183 23250747 PMC3567666

[25] Calzada E, Avery E, Sam PN, Modak A, Wang C, McCaffery JM, et al. Phosphatidylethanolamine made in the inner mitochondrial membrane is essential for yeast cytochrome bc1 complex function. Nat Commun. 2019;10(1):1432. doi: 10.1038/s41467-019-09425-1 30926815 PMC6441012

[26] Kawana H, Ozawa M, Shibata T, Onishi H, Sato Y, Kano K, et al. Identification and characterization of LPLAT7 as an sn-1-specific lysophospholipid acyltransferase. J Lipid Res. 2022;63(10):100271. doi: 10.1016/j.jlr.2022.100271 36049524 PMC9587406

[27] Jonas A. Lecithin cholesterol acyltransferase. Biochim Biophys Acta. 2000;1529(1–3):245–56. doi: 10.1016/s1388-1981(00)00153-0 11111093

[28] Satou M, Nishi Y, Yoh J, Hattori Y, Sugimoto H. Identification and characterization of acyl-protein thioesterase 1/lysophospholipase I as a ghrelin deacylation/lysophospholipid hydrolyzing enzyme. Endocrinology. 2010;151:4765–75. doi: 10.1210/en.2010-0412 20685872

[29] Herb M, Schramm M. Functions of ROS in Macrophages and Antimicrobial Immunity. Antioxidants (Basel). 2021;10(2):313. doi: 10.3390/antiox10020313 33669824 PMC7923022

[30] Flannagan RS, Heit B, Heinrichs DE. Antimicrobial Mechanisms of Macrophages and the Immune Evasion Strategies of Staphylococcus aureus. Pathogens. 2015;4(4):826–68. doi: 10.3390/pathogens4040826 26633519 PMC4693167

[31] Harada H, Becknell B, Wilm M, Mann M, Huang LJ, Taylor SS, et al. Phosphorylation and inactivation of BAD by mitochondria-anchored protein kinase A. Mol Cell. 1999;3(4):413–22. doi: 10.1016/s1097-2765(00)80469-4 10230394

[32] Bourens M, Fontanesi F, Soto IC, Liu J, Barrientos A. Redox and reactive oxygen species regulation of mitochondrial cytochrome C oxidase biogenesis. Antioxid Redox Signal. 2013;19(16):1940–52. doi: 10.1089/ars.2012.4847 22937827 PMC3852343

[33] Radin I, Kost L, Gey U, Steinebrunner I, Rödel G. The mitochondrial copper chaperone COX11 has an additional role in cellular redox homeostasis. PLoS One. 2021;16(12):e0261465. doi: 10.1371/journal.pone.0261465 34919594 PMC8682889

[34] Zhou M, Liu Y, Li C, Yang X, Ji C, Li W, et al. INSL3 promotes macrophage polarization to an immunosuppressive phenotype via the cAMP downstream signaling pathway and Akt/mTOR pathway. Int Immunopharmacol. 2025;154:114540. doi: 10.1016/j.intimp.2025.114540 40168802

[35] Patel HB, Montero-Melendez T, Greco KV, Perretti M. Melanocortin receptors as novel effectors of macrophage responses in inflammation. Front Immunol. 2011;2:41. doi: 10.3389/fimmu.2011.00041 22566831 PMC3342072

[36] Marin EP, Jozsef L, Di Lorenzo A, Held KF, Luciano AK, Melendez J, et al. The Protein Acyl Transferase ZDHHC21 Modulates α1 Adrenergic Receptor Function and Regulates Hemodynamics. Arterioscler Thromb Vasc Biol. 2016;36(2):370–9. doi: 10.1161/ATVBAHA.115.306942 26715683 PMC4984414

[37] Lin H. Protein cysteine palmitoylation in immunity and inflammation. FEBS J. 2021;288(24):7043–59. doi: 10.1111/febs.15728 33506611 PMC8872633

[38] Malhotra A, Ziegler A, Shu L, Perrier R, Amlie-Wolf L, Wohler E. De novo missense variants in LMBRD2 are associated with developmental and motor delays. J Med Genet. 2021;58:712–6. doi: 10.1136/jmedgenet-2020-107137 32820033 PMC11431178

[39] West AP, Brodsky IE, Rahner C, Woo DK, Erdjument-Bromage H, Tempst P, et al. TLR signalling augments macrophage bactericidal activity through mitochondrial ROS. Nature. 2011;472(7344):476–80. doi: 10.1038/nature09973 21525932 PMC3460538

[40] Starkov AA. The role of mitochondria in reactive oxygen species metabolism and signaling. Ann N Y Acad Sci. 2008;1147:37–52. doi: 10.1196/annals.1427.015 19076429 PMC2869479

[41] Lee H-J, Woo Y, Hahn T-W, Jung YM, Jung Y-J. Formation and Maturation of the Phagosome: A Key Mechanism in Innate Immunity against Intracellular Bacterial Infection. Microorganisms. 2020;8(9):1298. doi: 10.3390/microorganisms8091298 32854338 PMC7564318

[42] Gutiérrez S, Fischer J, Ganesan R, Hos NJ, Cildir G, Wolke M, et al. Salmonella Typhimurium impairs glycolysis-mediated acidification of phagosomes to evade macrophage defense. PLoS Pathog. 2021;17(9):e1009943. doi: 10.1371/journal.ppat.1009943 34555129 PMC8491875

[43] Flannagan RS, Kuiack RC, McGavin MJ, Heinrichs DE. Staphylococcus aureus Uses the GraXRS Regulatory System To Sense and Adapt to the Acidified Phagolysosome in Macrophages. mBio. 2018;9(4):e01143-18. doi: 10.1128/mBio.01143-18 30018109 PMC6050959

[44] Gao Y, Chen Y, Zhan S, Zhang W, Xiong F, Ge W. Comprehensive proteome analysis of lysosomes reveals the diverse function of macrophages in immune responses. Oncotarget. 2017;8(5):7420–40. doi: 10.18632/oncotarget.14558 28088779 PMC5352332

[45] Cummings RD. The mannose receptor ligands and the macrophage glycome. Curr Opin Struct Biol. 2022;75:102394. doi: 10.1016/j.sbi.2022.102394 35617912 PMC10243190

[46] Graham JB, Sunryd JC, Mathavan K, Weir E, Larsen ISB, Halim A. Endoplasmic reticulum transmembrane protein TMTC3 contributes to O-mannosylation of E-cadherin. Molecular Biology of the Cell. 2020;31:167–83. doi: 10.1091/mbc.E19-07-0408 31851597 PMC7001481

[47] Aquino RS, Park PW. Glycosaminoglycans and infection. Front Biosci (Landmark Ed). 2016;21(6):1260–77. doi: 10.2741/4455 27100505 PMC4975577

[48] Rostand KS, Esko JD. Microbial adherence to and invasion through proteoglycans. Infect Immun. 1997;65(1):1–8. doi: 10.1128/iai.65.1.1-8.1997 8975885 PMC174549

[49] Eisenhaber B, Sinha S, Jadalanki CK, Shitov VA, Tan QW, Sirota FL, et al. Conserved sequence motifs in human TMTC1, TMTC2, TMTC3, and TMTC4, new O-mannosyltransferases from the GT-C/PMT clan, are rationalized as ligand binding sites. Biol Direct. 2021;16(1):4. doi: 10.1186/s13062-021-00291-w 33436046 PMC7801869

[50] Liu J, Xiang J, Li X, Blankson S, Zhao S, Cai J, et al. NF-κB activation is critical for bacterial lipoprotein tolerance-enhanced bactericidal activity in macrophages during microbial infection. Sci Rep. 2017;7:40418. doi: 10.1038/srep40418 28079153 PMC5227741

[51] Gutierrez MG, Mishra BB, Jordao L, Elliott E, Anes E, Griffiths G. NF-kappa B activation controls phagolysosome fusion-mediated killing of mycobacteria by macrophages. J Immunol. 2008;181(4):2651–63. doi: 10.4049/jimmunol.181.4.2651 18684956

[52] Rahman MM, McFadden G. Modulation of NF-κB signalling by microbial pathogens. Nat Rev Microbiol. 2011;9(4):291–306. doi: 10.1038/nrmicro2539 21383764 PMC3611960

[53] Zhang X, Hu Y, Wang B, Yang S. Ferroptosis: Iron-mediated cell death linked to disease pathogenesis. J Biomed Res. 2024;38(5):413–35. doi: 10.7555/JBR.37.20230224 38808552 PMC11461536

[54] Saha P, Xiao X, Yeoh BS, Chen Q, Katkere B, Kirimanjeswara GS, et al. The bacterial siderophore enterobactin confers survival advantage to Salmonella in macrophages. Gut Microbes. 2019;10(3):412–23. doi: 10.1080/19490976.2018.1546519 30449241 PMC6546333

[55] Bastidas-Caldes C, de Waard JH, Salgado MS, Villacís MJ, Coral-Almeida M, Yamamoto Y, et al. Worldwide Prevalence of mcr-mediated Colistin-Resistance Escherichia coli in Isolates of Clinical Samples, Healthy Humans, and Livestock-A Systematic Review and Meta-Analysis. Pathogens. 2022;11(6):659. doi: 10.3390/pathogens11060659 35745513 PMC9230117

[56] Kaper JB, Nataro JP, Mobley HL. Pathogenic Escherichia coli. Nat Rev Microbiol. 2004;2(2):123–40. doi: 10.1038/nrmicro818 15040260

[57] Mukhopadhyay S, Plüddemann A, Hoe JC, Williams KJ, Varin A, Makepeace K, et al. Immune inhibitory ligand CD200 induction by TLRs and NLRs limits macrophage activation to protect the host from meningococcal septicemia. Cell Host Microbe. 2010;8(3):236–47. doi: 10.1016/j.chom.2010.08.005 20833375

[58] Behmoaras J, Bhangal G, Smith J, McDonald K, Mutch B, Lai PC, et al. Jund is a determinant of macrophage activation and is associated with glomerulonephritis susceptibility. Nat Genet. 2008;40(5):553–9. doi: 10.1038/ng.137 18443593 PMC2742200

[59] Koning N, van Eijk M, Pouwels W, Brouwer MSM, Voehringer D, Huitinga I, et al. Expression of the inhibitory CD200 receptor is associated with alternative macrophage activation. J Innate Immun. 2010;2(2):195–200. doi: 10.1159/000252803 20375636

[60] Berezhnoy NV, Cazenave-Gassiot A, Gao L, Foo JC, Ji S, Regina VR, et al. Transient Complexity of E. coli Lipidome Is Explained by Fatty Acyl Synthesis and Cyclopropanation. Metabolites. 2022;12(9):784. doi: 10.3390/metabo12090784 36144187 PMC9500627

[61] Herzig S, Shaw RJ. AMPK: guardian of metabolism and mitochondrial homeostasis. Nat Rev Mol Cell Biol. 2018;19(2):121–35. doi: 10.1038/nrm.2017.95 28974774 PMC5780224

[62] Blander JM, Medzhitov R. On regulation of phagosome maturation and antigen presentation. Nat Immunol. 2006;7(10):1029–35. doi: 10.1038/ni1006-1029 16985500

[63] Snider SA, Margison KD, Ghorbani P, LeBlond ND, O’Dwyer C, Nunes JRC, et al. Choline transport links macrophage phospholipid metabolism and inflammation. J Biol Chem. 2018;293(29):11600–11. doi: 10.1074/jbc.RA118.003180 29880645 PMC6065184

[64] Petkevicius K, Virtue S, Bidault G, Jenkins B, Çubuk C, Morgantini C. Accelerated phosphatidylcholine turnover in macrophages promotes adipose tissue inflammation in obesity. eLife. 2019;8:e47990. doi: 10.7554/eLife.47990 PMC6748830 31418690

[65] Das D, Bishayi B. Contribution of Catalase and Superoxide Dismutase to the Intracellular Survival of Clinical Isolates of Staphylococcus aureus in Murine Macrophages. Indian J Microbiol. 2010;50(4):375–84. doi: 10.1007/s12088-011-0063-z 22282603 PMC3209836

[66] Wang Y, Jiao R, Zhang X, Ren Y, Zhao W, Ye Y. OmpR-mediated activation of the type Vl secretion system drives enhanced acid tolerance in Cronobacter. J Dairy Sci. 2025;108(4):3390–403. doi: 10.3168/jds.2024-25685 39890079

[67] Andrews SC, Robinson AK, Rodríguez-Quiñones F. Bacterial iron homeostasis. FEMS Microbiol Rev. 2003;27(2–3):215–37. doi: 10.1016/S0168-6445(03)00055-X 12829269

[68] Kwun MS, Lee DG. Ferroptosis-Like Death in Microorganisms: A Novel Programmed Cell Death Following Lipid Peroxidation. J Microbiol Biotechnol. 2023;33(8):992–7. doi: 10.4014/jmb.2307.07002 37463851 PMC10471485

[69] Valvano MA. Remodelling of the Gram-negative bacterial Kdo2-lipid A and its functional implications. Microbiology (Reading). 2022;168(4):10.1099/mic.0.001159. doi: 10.1099/mic.0.001159 35394417

[70] Hohle TH, Franck WL, Stacey G, O’Brian MR. Bacterial outer membrane channel for divalent metal ion acquisition. Proc Natl Acad Sci U S A. 2011;108(37):15390–5. doi: 10.1073/pnas.1110137108 21880957 PMC3174606

[71] Ma W, Jiang X, Dou Y, Zhang Z, Li J, Yuan B, et al. Biophysical Impact of Lipid A Modification Caused by Mobile Colistin Resistance Gene on Bacterial Outer Membranes. J Phys Chem Lett. 2021;12(48):11629–35. doi: 10.1021/acs.jpclett.1c03295 34817187

[72] Schumann A, Gaballa A, Yang H, Yu D, Ernst RK, Wiedmann M. Site-selective modifications by lipid A phosphoethanolamine transferases linked to colistin resistance and bacterial fitness. mSphere. 2024;9(12):e0073124. doi: 10.1128/msphere.00731-24 39611852 PMC11656738

[73] Yang QE, MacLean C, Papkou A, Pritchard M, Powell L, Thomas D, et al. Compensatory mutations modulate the competitiveness and dynamics of plasmid-mediated colistin resistance in Escherichia coli clones. ISME J. 2020;14(3):861–5. doi: 10.1038/s41396-019-0578-6 31896787 PMC7031280

[74] Geng J, Sun X, Wang P, Zhang S, Wang X, Wu H, et al. Kinases Mst1 and Mst2 positively regulate phagocytic induction of reactive oxygen species and bactericidal activity. Nat Immunol. 2015;16(11):1142–52. doi: 10.1038/ni.3268 26414765 PMC4618176

[75] Breyer F, Härtlova A, Thurston T, Flynn HR, Chakravarty P, Janzen J, et al. TPL-2 kinase induces phagosome acidification to promote macrophage killing of bacteria. EMBO J. 2021;40(10):e106188. doi: 10.15252/embj.2020106188 33881780 PMC8126920

[76] Palacin-Martinez C, Anel-Lopez L, Alvarez M, Neila-Montero M, Montes-Garrido R, Soriano-Úbeda C, et al. The characterization of CellROX™ probes could be a crucial factor in ram sperm quality assessment. Front Vet Sci. 2024;11:1342808. doi: 10.3389/fvets.2024.1342808 38476170 PMC10927726

[77] Cabrera-Wrooman A, Ortega-Peña S, Salgado RM, Sandoval-Cuevas B, Krötzsch E. Antiseptic Effects and Biosafety of a Controlled-Flow Electrolyzed Acid Solution Involve Electrochemical Properties, Rather than Free Radical Presence. Microorganisms. 2022;10(4):745. doi: 10.3390/microorganisms10040745 35456795 PMC9032035

[78] Rodríguez-Rojas A, Kim JJ, Johnston PR, Makarova O, Eravci M, Weise C, et al. Non-lethal exposure to H2O2 boosts bacterial survival and evolvability against oxidative stress. PLoS Genet. 2020;16(3):e1008649. doi: 10.1371/journal.pgen.1008649 32163413 PMC7093028

