# Supplementary material for: Macrophage-mediated antibiotic evasion and competitive dominance of mcr-3-carrying Escherichia coli
Source: PLoS Pathog. 2026 Jul 14;22(7):e1014427. doi: 10.1371/journal.ppat.1014427 (PMC13387617; doi:10.1371/journal.ppat.1014427)
Supplement: S1 File — Original uncropped and unadjusted images underlying all blot results. (A) Original images for P65, p-P65, and GAPDH blots corresponding to Fig 5H. (B) Original images for GPX4, COX2, and GAPDH blots corresponding to Fig 6D. Molecular weights of the detected proteins are indicated on the right. Where applicable, membranes were cut before antibody incubation or imaging, and the full captured image of each resulting membrane strip is shown. (PDF) [file ppat.1014427.s009.pdf]

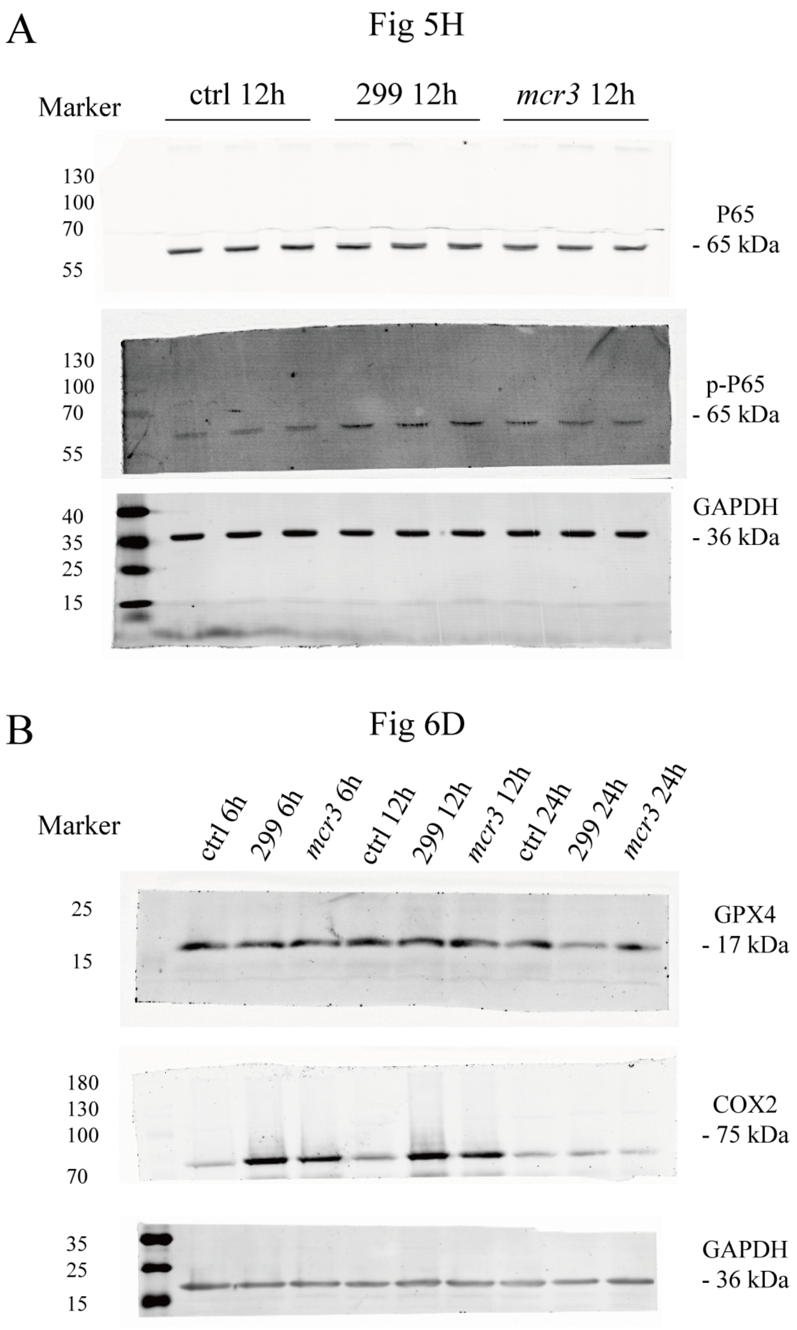


**S1 Raw Images. Original uncropped and unadjusted images underlying all blot results.** (A) Original images for P65, p-P65, and GAPDH blots corresponding to Fig 5H. (B) Original images for GPX4, COX2, and GAPDH blots corresponding to Fig 6D. Molecular weights of the detected proteins are indicated on the right. Where applicable, membranes were cut before antibody incubation or imaging, and the full captured image of each resulting membrane strip is shown.
